# Supplementary material for: Complementary roles of serotonergic and cholinergic systems in decisions about when to act
Source: Curr Biol. 2022 Mar 14;32(5):1150–1162.e7. doi: 10.1016/j.cub.2022.01.042 (PMC8926843; doi:10.1016/j.cub.2022.01.042)
Supplement: Document S1. Figures S1–S6 and Tables S1 and S2 [file mmc1.pdf]

Current Biology, Volume *32*

Supplemental Information

Complementary roles of serotonergic and cholinergic systems in decisions about when to act

Nima Khalighinejad, Sanjay Manohar, Masud Husain, and Matthew F.S. Rushworth

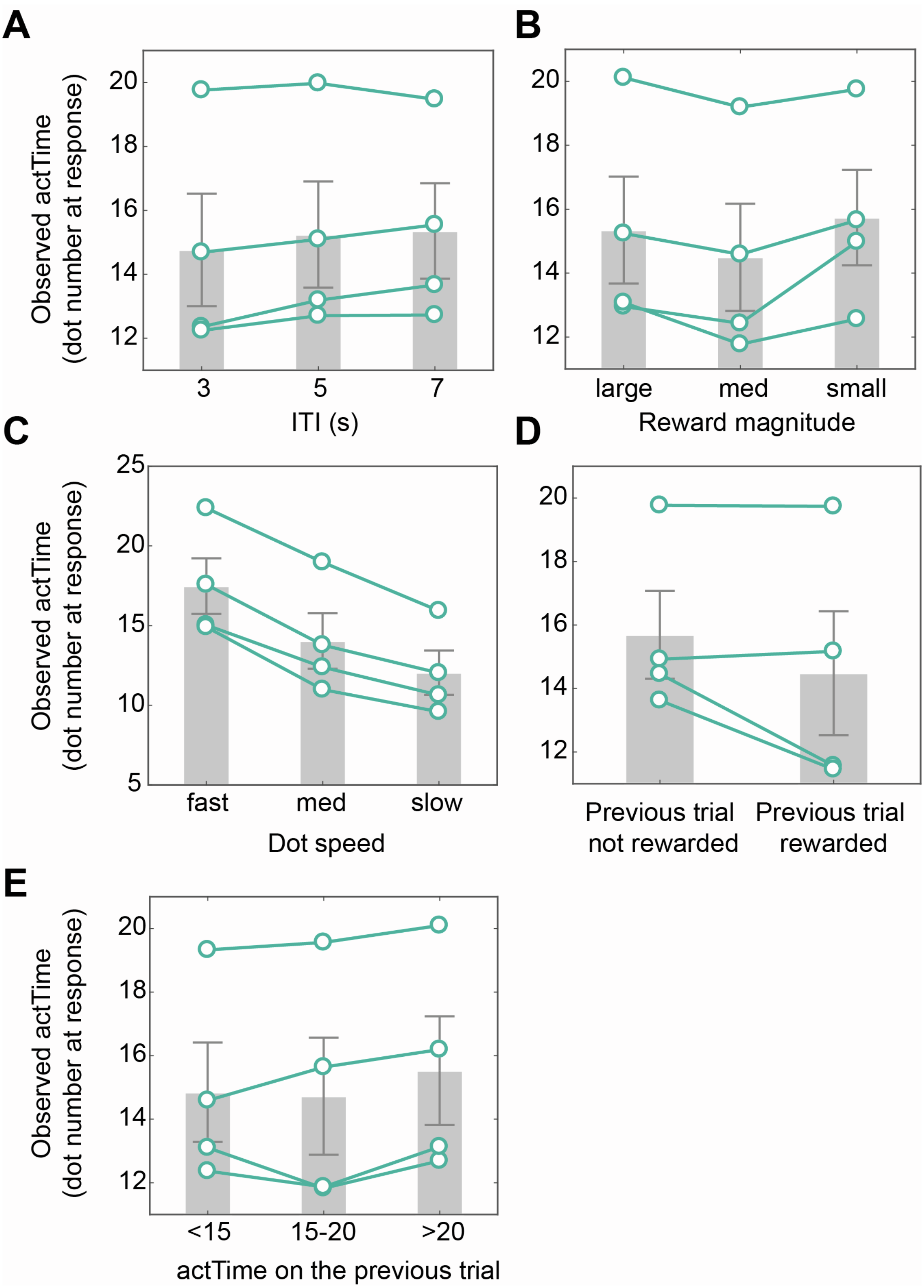

A
B
C
D
E
Observed actTime (dot number at response)
ITI (s)
3
5
7
Reward magnitude
large
med
small
Dot speed
fast
med
slow
Previous trial not rewarded
Previous trial rewarded
actTime on the previous trial
<15
15-20
>20

Figure S1. The relationship between observed *actTime* and experimentally manipulated variation in the present and recent past contextual factors. Related to Figure 1. This relationship was assessed using the same method as in a previous study[S1]. A multilevel ANOVA (STAR Methods; Khalighinejad et al., 2020) showed that all aspects of present context including ITI (A) ($X^2(2)=17$, $P<0.001$); reward magnitude (B) ($X^2(2)=62$, $P<0.001$); dot speed (C) ($X^2(2)=939$, $P<0.001$); past context including reward outcome on past trial (D) ($X^2(3)=231$, $P<0.001$); and *actTime* on past trial (E) ($X^2(1)=27$, $P<0.001$) influenced animals' *actTime* on the current trial. Post-hoc Tukey's HSD tests showed that animals waited longer before making a response during long compared to short ITI blocks ($\beta=0.14\pm0.03$, $Z=4.14$), when offered a small compared to medium reward ($\beta=0.30\pm0.04$, $Z=8.20$), in fast compared to slow dot speed condition ($\beta=1.24\pm0.03$, $Z=42$), when they had received no reward compared to a large reward on the past trial ($\beta=0.48\pm0.03$, $Z=15$), and when they had already delayed *actTime* on the past trial ($\beta=0.07\pm0.01$). The grey columns are the mean across animals, error bars are the standard error of the mean across animals, and each line is data from individual animals. For illustrative purposes, *actTime* in the immediate past trial is binned into three groups.

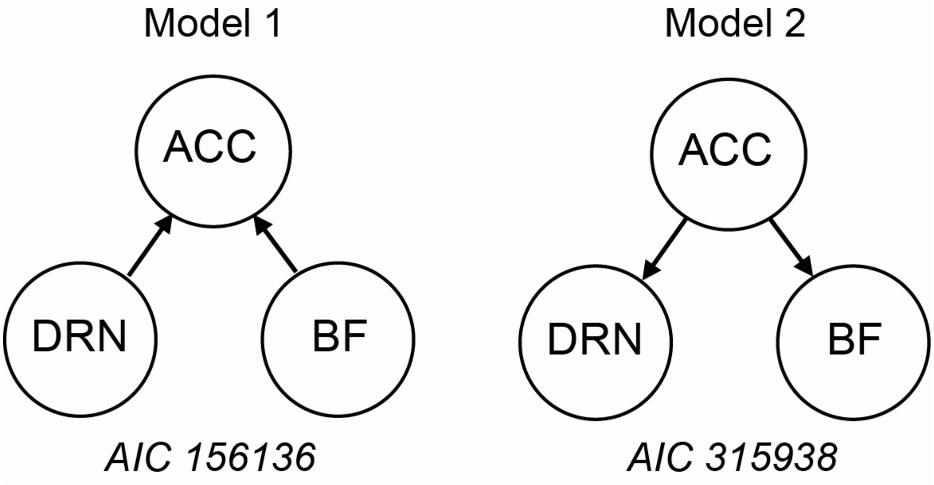

Figure S2. Effective connectivity between ACC, DRN and BF. Related to Figure 3G. Even though PPI analyses demonstrate functional coupling between ROIs as a function of specific psychological measures it does not show directionality. We therefore performed structural equation modelling (SEM) to investigate the direction of the effects. We compared two plausible models: Model 1 has directional connection from DRN and BF to ACC. We did not have any prediction about the directionality of the effect between BF and DRN and therefore did not assume any directionality. Model 2 was similar to Model 1, but all the directions were reversed. SEM showed that a model in which activity in BF and DRN influence ACC (Model1 AIC: 156136) is a better fit to the data than the alternative model (Model2 AIC: 315938).

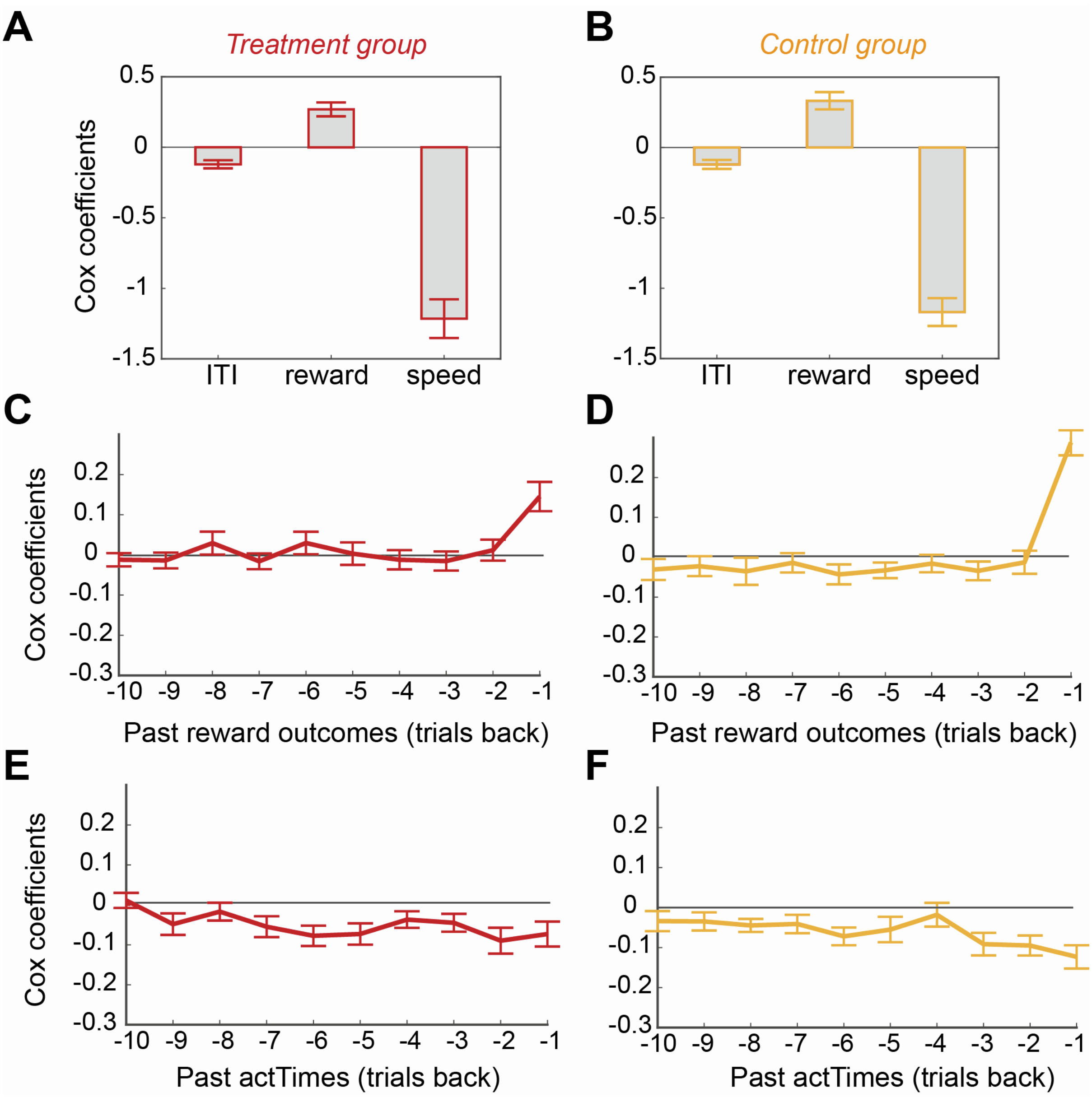

A
Treatment group
Cox coefficients
0.5
0
-0.5
-1
-1.5
ITI
reward
speed
B
Control group
0.5
0
-0.5
-1
-1.5
ITI
reward
speed
C
Cox coefficients
0.2
0.1
0
-0.1
-0.2
-0.3
-10 -9 -8 -7 -6 -5 -4 -3 -2 -1
Past reward outcomes (trials back)
D
0.2
0.1
0
-0.1
-0.2
-0.3
-10 -9 -8 -7 -6 -5 -4 -3 -2 -1
Past reward outcomes (trials back)
E
Cox coefficients
0.2
0.1
0
-0.1
-0.2
-0.3
-10 -9 -8 -7 -6 -5 -4 -3 -2 -1
Past actTimes (trials back)
F
0.2
0.1
0
-0.1
-0.2
-0.3
-10 -9 -8 -7 -6 -5 -4 -3 -2 -1
Past actTimes (trials back)

Figure S3. Coefficients for the covariates used in the Cox regression model. Related to Figure 4. To estimate the fraction of the observed *actTime* that could be predicted from immediate contextual factors we used a Cox proportional hazard model (see STAR Methods). The model predicted time-to-event (*actTime*) on the current trial as a function of the immediate recent past and present context. Specifically, the predictors included reward magnitude, dot speed, and ITI of the current trial (A,B), and the actual reward (C,D) and *actTime* (E,F) on the past 10 trials. The coefficients were estimated separately for each testing session and were used to measure the trial-by-trial variation in *actTime* (i.e., *deterministic actTime*). Serotonergic manipulation had no significant effect on *deterministic actTime* as predicted from the immediate context (Figure 4B). Interestingly, ITI – as compared to other more explicit, stimulus-based features of the environment such as the reward magnitude and dot speed – had the smallest coefficient and therefore a negligible effect on trial-by-trial variation in *deterministic actTime*. This could be due to the fact that ITI – unlike other features of the immediate, present context that varied from trial-to-trial – changed in blocks of 30 trials. This could also explain why ITI's influence on *actTime* and DRN BOLD (see Figures S1 & 5) was comparable to that of the environment's average value (see Figures 1H & 3B). Similarly, the effects of the past reward outcomes and the past *actTimes* were stronger in the immediately recent past trials compared to distant past trials. This further supports our assumption that trial-by trial variation in *actTime* is mostly driven by stimulus-based, immediate, present and recent past context, while ITI and distant past have a more tonic, slow-changing effect on *actTime*, similar to that of the broader, general environment. Error bars are the standard error of the mean across testing sessions.

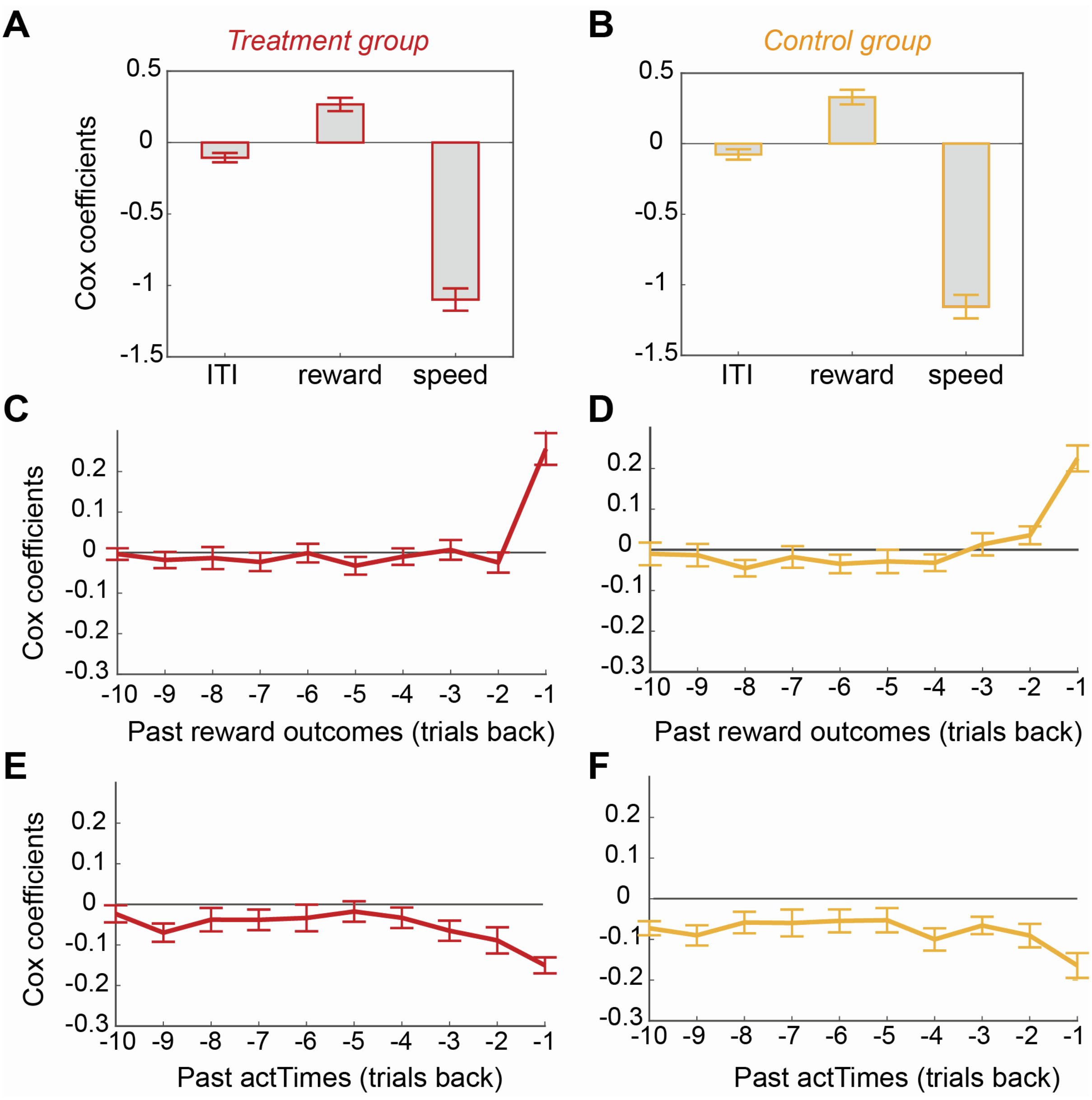
A
Treatment group
B
Control group
Cox coefficients
0.5
0
-0.5
-1
-1.5
ITI
reward
speed
C
D
0.2
0.1
0
-0.1
-0.2
-0.3
-10
-9
-8
-7
-6
-5
-4
-3
-2
-1
Past reward outcomes (trials back)
E
F
Past actTimes (trials back)

Figure S4. Coefficients for the covariates used in the Cox regression model. Related to Figure 6. Similar to Figure S3 but related to data from Exp.3 (cholinergic manipulation). The coefficients were estimated separately for each testing session and were used to measure the trial-by-trial variation in *actTime* (i.e., *deterministic actTime*). Cholinergic manipulation significantly reduced the length of time each animal was expected to wait on each trial before making a response, as predicted from the combined effect of the immediate recent past and present context (i.e., *deterministic actTime;* Figure 6B). However, comparing the coefficients between the Treatment and the Control groups showed that while the combined effect of the contextual factors reduced *deterministic actTime* in the Treatment group, this effect was not mediated by any one particular factor. This is consistent with the interaction effects in GLM3.1. Additionally, looking at Figure 6 it seems that in one particular animal (Monkey C) the effect of cholinergic manipulation has opposite effects on observed and deterministic *actTime*. We think this difference is due to two reasons: First, it stems from the relatively high number of un-responded trials in Monkey C (Figure 6C; %30). This is problematic for the observed *actTime* analysis because it leads to a reduction in data. However, it is less problematic for the deterministic *actTime* analysis because these missed trials are modelled as censored data in the Cox regression model (i.e., the event time exceeds the censoring time). Second, the deterministic *actTime* in Monkey C has an ex-Gaussian distribution with a relatively long tail, similar to distribution of the observed and deterministic *actTime* in other three monkeys, but different from the large-width Gaussian distribution of observed *actTime* in the same monkey. The effect of cholinergic manipulation on *actTime* could therefore be more easily identified as a change in the mean of the Gaussian component of the ex-Gaussian distribution in this monkey. It thus became clear, when using this approach, that a similar change had occurred in all four animals under the cholinergic manipulation. Error bars are the standard error of the mean across testing sessions.

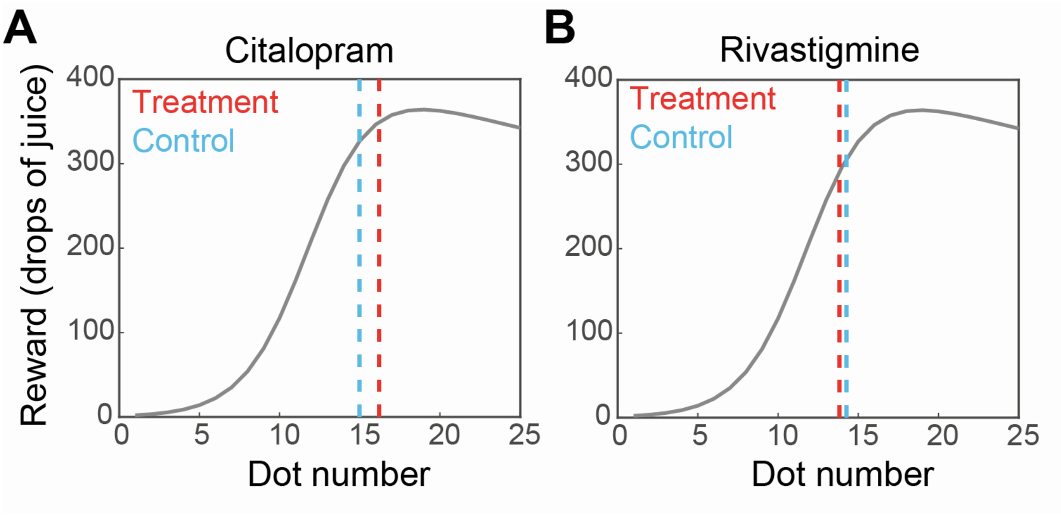

Figure S5. The effect of citalopram and rivastigmine on accumulated reward. Related to Figures 4&6. While our approach does not provide a normative account of animals' decisions it is possible to estimate the best speed on which to respond given the various task features such as the sigmoid reward function (Figure 1C) and the total length of each session (40 minutes). This shows that monkeys would collect the highest amount of reward (drops of juice) if they waited for 19 dots before responding. In the Citalopram study (A) monkeys on average waited for 16.2 dots before responding in the Treatment condition (red dashed line) and 15.2 dots in the Control condition (blue dashed line). This resulted in monkeys collecting significantly more reward in the Treatment compared to the Control group ($t(19)=2.36$, $P=0.029$). When we make an analogous comparison in the Rivastigmine study (B), however, we found no significant difference between the reward rates in the Treatment and the Control conditions ($P=0.51$). This suggests that in healthy animals increasing levels of ACh does not make the behaviour more optimal. However, in pathologies such as Parkinson's disease, which is often associated with apathy, rivastigmine might help to alleviate symptoms by invigorating volitional movements in response to environmental stimuli.

The slowing of *actTime* induced by SSRI administration led the animals to respond more closely to the optimum time and so they obtained more rewards in total. Some of the changes in *actTime* that occurred when the task environment was changed in the absence of any pharmacological treatment may also have been adaptive. For example, macaques responded more deliberately and slowly during long compared to short ITI blocks when the rate of reward was lower than the average reward rate elsewhere in the same day's testing session; careful, long *actTimes* ensure opportunities to obtain reward are not wasted before a long transition to the next trial. However, the finding that animals responded more slowly on medium value trials that occurred in the context of high average value, is not clearly

adaptive in the same way. Slow responses on such trials entail an opportunity cost; the opportunity costs of acting slowly are higher when the offer is worth less than the average value of the environment. A full account of how and why changes in reward distributions lead to changes in vigour and speed of responding remains elusive.

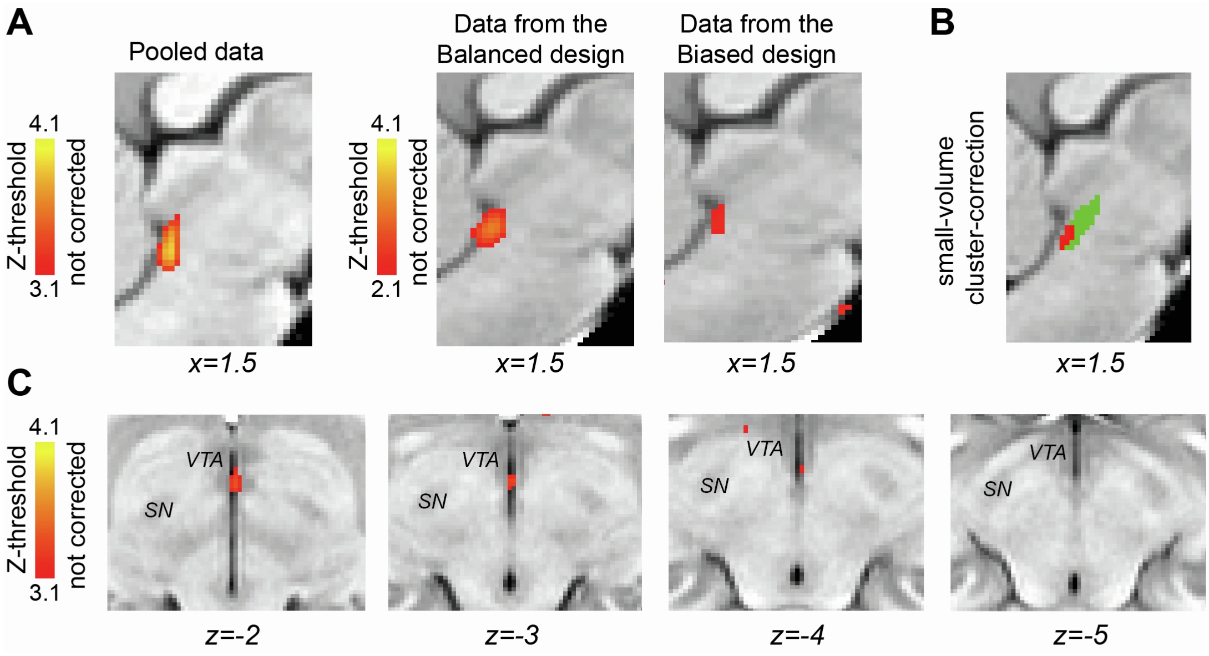

Figure S6. The relationship between BOLD and ITI. Related to Figure 5. (A) Correlation between DRN BOLD and ITI shown separately for the pooled data, data from the balanced design, and data from the biased design. the ITI effect at DRN was weaker when looking at the datasets separately (because each analysis employs approximately half as much data as is the case when the data sets are combined) and could only be detected at lower Z-thresholds (Z>2.1). Nevertheless, in both datasets we found a positive correlation between DRN voxels and ITI. (B) The effect of ITI from the DRN region that was used in Exp.1 for the analysis of the average value. The green mask shows the DRN region that was used in Exp.1. The overlapping red mask shows the cluster with a significant ITI effect (Z-threshold=3.1; peak Z=3.82, Caret-F99 Atlas (F99): x=1.0, y=-21, z=-8.5; small-volume correction; number of voxels=31, P=0.003). (C) The volume of interest that we used in Figure 5 already covered the midbrain including the dopaminergic structures that are often associated with encoding of the reward rate including the ventral tegmental area (VTA) and the substantia nigra (SN). Having found no significant cluster other than DRN thus suggests that dopaminergic midbrain activity is not significantly correlated with ITI. However, it is still possible that without cluster correction we might detect an ITI effect in dopaminergic midbrain. Therefore, without performing cluster correction, we searched for voxels within VTA/SN but could not find any with significant positive correlation with ITI (Z>3.1).

	**Monkey 1**	**Monkey 2**	**Monkey 3**	**Monkey 4**
Phase I				
**Day 1-7 (10mg)**	*Placebo*	*Placebo*	*Citalopram*	*Citalopram*
**Day 8-14 (20mg)**	*Placebo*	*Placebo*	*Citalopram*	*Citalopram*
**Day 15-24 (20mg)**	*Placebo*	*Placebo*	*Citalopram*	*Citalopram*
**5-HT measurement (nmoles/L)**	1519	1780	70	103
**Day 25-38 (Wash-out)**				
Phase II				
**Day 39-45 (10mg)**	*Citalopram*	*Citalopram*	*Placebo*	*Placebo*
**Day 46-52 (20mg)**	*Citalopram*	*Citalopram*	*Placebo*	*Placebo*
**Day 53-62 (20mg)**	*Citalopram*	*Citalopram*	*Placebo*	*Placebo*
**5-HT measurement (nmoles/L)**	160	280	1863	1137

Table S1. Citalopram dosing schedule. Related to Figure 4. Behavioural data collection for the main analyses was conducted on alternate days during the last 10 days (green cells). Groups were switched after a 2-week wash-out period. 5-HT levels were measured in the platelet rich plasma (PRP) after each treatment phase (within-subject comparison). Note that selective serotonin reuptake inhibitors block serotonin receptors (SERT) on platelets, thereby preventing reuptake of 5-HT into platelets. The results clearly show that concentration of 5-HT within platelets decreased when monkeys were in the treatment compared to the control group. We also collected blood samples after ACh treatment to check that the manipulations were working. However, measurement of ACh levels for all individuals was not possible because of the impact of COVID-19-related lockdown in the UK.

	**Monkey 1**	**Monkey 2**	**Monkey 3**	**Monkey 4**
Phase I				
**Day 1-7 (0.37mg)**	*Rivastigmine*	*Rivastigmine*	*Placebo*	*Placebo*
**Day 8-14 (0.75mg)**	*Rivastigmine*	*Rivastigmine*	*Placebo*	*Placebo*
**Day 15-24 (1.5mg)**	*Rivastigmine*	*Rivastigmine*	*Placebo*	*Placebo*
**Day 25-38 (Wash-out)**				
Phase II				
**Day 39-45 (0.37mg)**	*Placebo*	*Placebo*	*Rivastigmine*	*Rivastigmine*
**Day 46-52 (0.75mg)**	*Placebo*	*Placebo*	*Rivastigmine*	*Rivastigmine*
**Day 53-62 (1.5mg)**	*Placebo*	*Placebo*	*Rivastigmine*	*Rivastigmine*

Table S2. Rivastigmine dosing schedule. Related to Figure 6. Behavioural data collection for the main analyses was conducted on alternate days during the last 10 days (green cells). Groups were switched after a 2-week wash-out period.

Supplemental References

S1. Khalighinejad, N., Bongioanni, A., Verhagen, L., Folloni, D., Attali, D., Aubry, J.-F., Sallet, J., and Rushworth, M.F.S. (2020). A Basal Forebrain-Cingulate Circuit in Macaques Decides It Is Time to Act. Neuron *105*, 370-384.e8.
