# Supplementary material for: Complementary roles of serotonergic and cholinergic systems in decisions about when to act
Source: Curr Biol. 2022 Mar 14;32(5):1150–1162.e7. doi: 10.1016/j.cub.2022.01.042 (PMC8926843; doi:10.1016/j.cub.2022.01.042)
Supplement: Document S2. Article plus supplemental information [file mmc2.pdf]

Article

Current Biology

Complementary roles of serotonergic and cholinergic systems in decisions about when to act

Highlights

- Both immediate context and wider environment influence decisions about when to act
- DRN and 5-HT mediate the influence of wider environment
- BF and ACh mediate the influence of immediate context

Authors

Nima Khalighinejad, Sanjay Manohar, Masud Husain, Matthew F.S. Rushworth

Correspondence

nima.khalighinejad@psy.ox.ac.uk

In brief

By using functional imaging and pharmacological manipulation, Khalighinejad et al. show that, although basal forebrain and cholinergic system modulate decisions about when to act by mediating the influence of specific aspects of immediate context on behavior, dorsal raphe nucleus and serotonergic system mediate the influence of the wider environment.

Khalighinejad et al., 2022, Current Biology *32*, 1150–1162
March 14, 2022 © 2022 The Authors. Published by Elsevier Inc.
https://doi.org/10.1016/j.cub.2022.01.042

CellPress

CellPress
OPEN ACCESS

Current Biology

Article

Complementary roles of serotonergic and cholinergic systems in decisions about when to act

Nima Khalighinejad,[1,4,5,*] Sanjay Manohar,[2,3] Masud Husain,[2,3] and Matthew F.S. Rushworth[1]
[1]Wellcome Centre for Integrative Neuroimaging, Department of Experimental Psychology, University of Oxford, Oxford, UK
[2]Department of Experimental Psychology, University of Oxford, Oxford, UK
[3]Nuffield Department of Clinical Neurosciences, University of Oxford, Oxford, UK
[4]Twitter: @nima_khalighi
[5]Lead contact
*Correspondence: nima.khalighinejad@psy.ox.ac.uk
https://doi.org/10.1016/j.cub.2022.01.042

SUMMARY

Decision-making not only involves deciding about which action to choose but when and whether to initiate an action in the first place. Macaque monkeys tracked number of dots on a screen and could choose when to make a response. The longer the animals waited before responding, the more dots appeared on the screen and the higher the probability of reward. Monkeys waited longer before making a response when a trial's value was less than the environment's average value. Recordings of brain activity with fMRI revealed that activity in dorsal raphe nucleus (DRN)—a key source of serotonin (5-HT)—tracked average value of the environment. By contrast, activity in the basal forebrain (BF)—an important source of acetylcholine (ACh)—was related to decision time to act as a function of immediate and recent past context. Interactions between DRN and BF and the anterior cingulate cortex (ACC), another region with action initiation-related activity, occurred as a function of the decision time to act. Next, we performed two psychopharmacological studies. Manipulating systemic 5-HT by citalopram prolonged the time macaques waited to respond for a given opportunity. This effect was more evident during blocks with long inter-trial intervals (ITIs) where good opportunities were sparse. Manipulating systemic acetylcholine (ACh) by rivastigmine reduced the time macaques waited to respond given the immediate and recent past context, a pattern opposite to the effect observed with 5-HT. These findings suggest complementary roles for serotonin/DRN and acetylcholine/BF in decisions about when to initiate an action.

INTRODUCTION

Tarsiers are pocket-size primates but ruthless ambush predators. To hunt they gather information from their environment using their disproportionately large eyes and ultrasonic hearing. They integrate this information with their past hunting experience and decide when to strike to have the highest chance of success. This sit-and-wait strategy is common among animal species, including humans. For example, an art collector may choose to bid for a specific item in an auction, but it is also important to place the bid at the right moment. Similarly, the tarsier may choose to ambush a desirable prey, but her strategy will fail if the surprise attack is launched at the wrong moment. Previous research on decision-making has often emphasized brain processes for choosing among action alternatives. However, decisions about "when" to initiate an action have attracted less attention.[1] This is important because impairments in decisions about if and when to act are observed across a wide range of brain disorders, such as apathy and impulsivity.[2,3]

The aim of this study was to establish what decisions about "when" to act—in non-human primates (NHPs)—depend upon. We predicted that such decisions depend not just on immediate context and consequences but additionally, on broader, general features of the environment beyond the current trial, such as its richness—how good opportunities are on average. In addition, we predicted that dorsal raphe nucleus (DRN) and basal forebrain (BF), major sources of serotonin (5-HT) and acetylcholine (ACh) in the brain,[4–6] mediate the influence of the broader environment and immediate context, respectively, on decisions about when to act. We made this prediction because tracking the average value of the environment and the immediate context has been linked to DRN and BF, respectively.[7,8] We investigated these hypotheses using functional magnetic resonance imaging (fMRI) and pharmacological manipulations. We identify central and complementary roles for, on the one hand, the DRN and 5-HT and, on the other hand, BF and ACh in controlling decision time to act by integrating distinct sources of information in animals' environments.

RESULTS

The average value of the environment influences animals' time to act

In the first experiment (Experiment 1), we investigated whether decisions about "when" to act depend not only on immediate

 © 2022 The Authors. Published by Elsevier Inc.
This is an open access article under the CC BY license (http://creativecommons.org/licenses/by/4.0/).

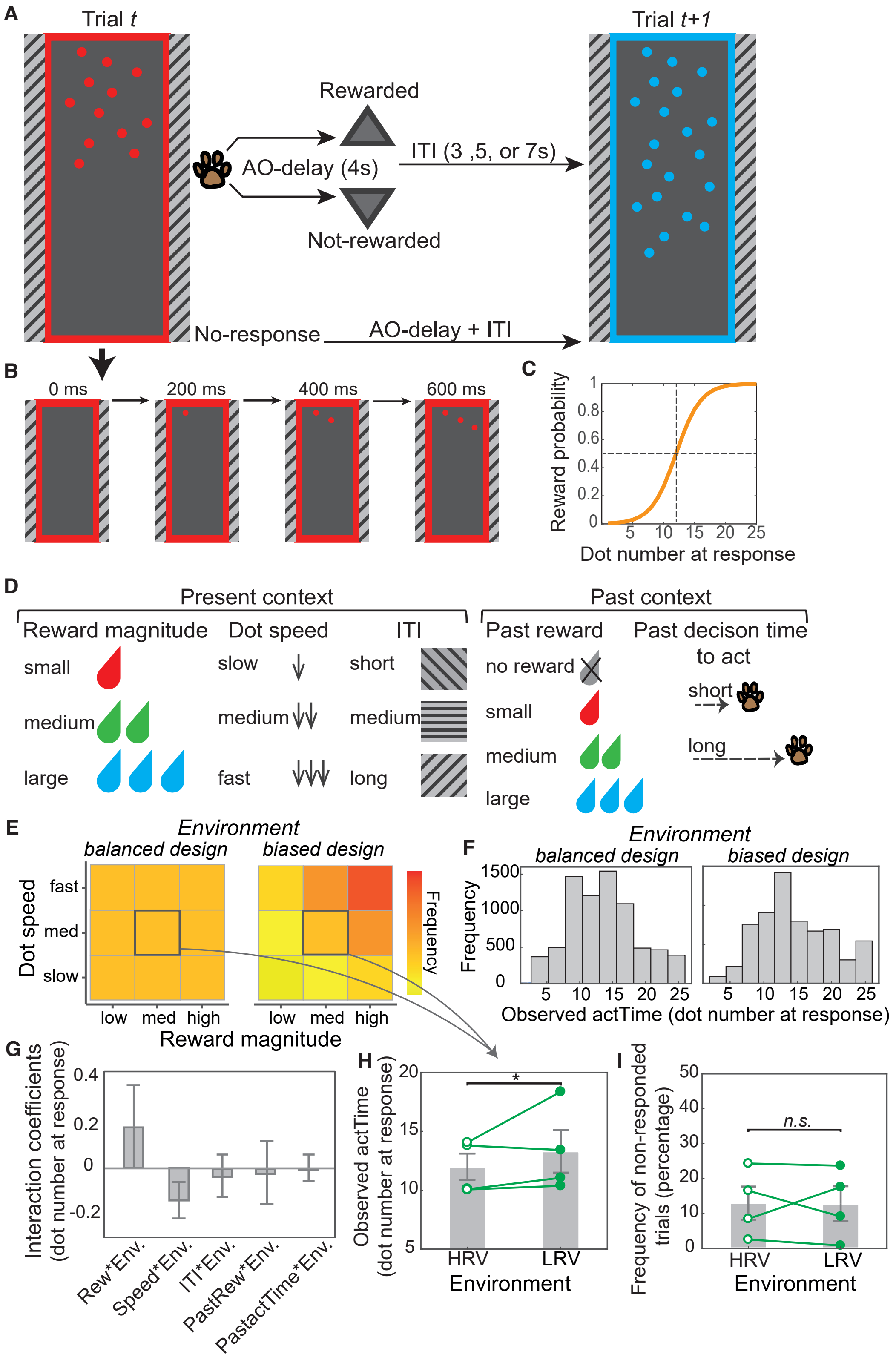

Figure 1. Experimental task and behavioral results

(A) Animals tracked the number of dots on the screen and responded by touching a response pad at a time of their choice. The color of the frame and the dots represented the potential reward magnitude on that trial. The patterns on each side of the screen represented ITI duration.

(legend continued on next page)

context and consequences but additionally on broader, general features of the environment beyond the current trial. To assess the effect of the environment on action time (*actTime*), we modified an experimental task that monkeys had previously been trained on.[7] Dots appeared one at a time on a screen. Animals tracked the number of dots and could choose when to make a response, by tapping on a response pad in front of them (Figures 1A and 1B; STAR Methods). The number of dots on the screen at the time of response determined the probability of reward, which was drawn from a sigmoid function: the longer the animals waited before responding, the more dots appeared on the screen and the higher the probability of reward (Figure 1C). This probability distribution remained constant across trials and sessions. Although impulsive responses were unlikely to yield reward, there was not much to gain from waiting for all dots to appear, given that the length of each testing session was limited to 40 min; therefore, there would be an opportunity cost from waiting too long.

We manipulated features of the immediate context known to influence animals' decisions about when to act[7] (Figure 1D). Three features determined the "immediate, present context": reward magnitude on the current trial, speed of the sequential appearance of the dots on the current trial, and inter-trial interval (ITI) prior to the current trial. Two features determined the "immediate, recent past context": the animal's own recent behavior and recent reward experience—the outcome and action time on the past trial. In addition, to investigate the effect of the "broader, general environment" on *actTime*, we manipulated the distribution of the offers: In the original design[7]—which we refer to as the "balanced" design—there were equal numbers of good offers (trials with high reward magnitude and fast dot speed), medium offers, and bad offers (trials with low reward magnitude and slow dot speed). Now, however, in Experiment 1 we increased the proportion of "good offers" and reduced the proportion of "bad offers"; we refer to this as the "biased" design (STAR Methods). Note, however, that there were equal numbers of "medium offer" trials in both designs but the relative value of the medium offer trials in comparison to average value of trials was lower in the biased, compared with the balanced design (Figure 1E). We exploited this discrepancy between the value of a "medium offer" trial and the environment to compare the effect of the "average value of the environment" on animals' behavior and brain activity. Accordingly, if the average value of the environment influenced animals' decisions about when to act, we would expect a difference in *actTime* on "medium offer" trials between the two environments.

On average, in the biased design, animals waited for 15 ± 3 dots before responding (n = 4; across 43 sessions), which was associated with an 82% chance of reward. This is comparable with the average *actTime* in the balanced design (14 ± 4; Figure 1F). Additionally, similar to previous findings,[7] observed *actTime* was influenced by all aspects of the experimentally manipulated factors that determined both "the immediate, present context" and "immediate, recent past context" (all $p < 0.001$; Figure S1 for full results). The effects of the "immediate, present context" and "immediate, recent past context" on observed *actTime* were not significantly different in the biased and balanced designs (all $\chi^2(1) < 3.18$; all $p > 0.074$; STAR Methods; GLM1.1; Figure 1G).

Next, to determine the effect of "broader, general context" we compared observed *actTime* across "medium offer" trials: monkeys waited longer before responding on "medium offer" trials in the biased design compared with the balanced design (mixed-effect model; GLM1.2; STAR Methods; $\beta = 0.68$, $\chi^2(1) = 6.35$, $p = 0.012$; Figure 1H). This suggests that action time was delayed on trials in which the value of the offer was worth less than the average value of the environment. This effect was specific to "medium" offer trials: no significant difference was found in *actTime* when only comparing across "good" offer ($p = 0.18$) or "bad" offer ($p = 0.58$) trials. Finally, we tested whether this effect could be explained by differences in the animals' overall engagement with the task by comparing the number of missed "medium offer" trials (trials on which animals did not make a response) between the biased and balanced designs. There was no significant difference ($\chi^2(1) = 0.32$, $p = 0.57$; Figure 1I). In summary, trial-by-trial variance in the observed *actTime* depends not only on "immediate past" and "present context" but additionally on the "broader, general environment" beyond the current trial.

DRN and BF mediate the influence of broader, general features of the environment and the immediate context on animals' time to act

The brain activity of monkeys was recorded with fMRI (43 scanning sessions; 11 scans/monkey except M1 with 10 scans) while they were performing the behavioral task. We have previously shown that anterior cingulate cortex (ACC) and BF—containing the medial septum/diagonal band of Broca—tracked trial-by-trial variation in the observed *actTime*.[7] First, we sought to

(B) Timeline of one trial. An empty frame appeared on the left or right side of the screen. It was gradually filled with dots emerging from top to bottom appearing every 100, 200, or 300 ms, depending on trial type.

(C) The probability of getting reward increased as more dots appeared on the screen, following a sigmoid curve.

(D) Features of the immediate present and recent past context predicted animals' *actTime*.

(E) Compared with a balanced design[7] (left panel), there were more high reward magnitude and fast dot speed offers in the biased (right panel) design. The relative value of "medium offer" trials (thick box border) was therefore higher in the balanced design.

(F) Distribution of observed *actTime* across all trials in data collected using balanced and biased designs.

(G) The effect of the immediate context on *actTime* was not statistically different between environments (Figure S1 illustrates main effect of immediate context on *actTime*). The interaction coefficients from GLM1.1. *Env.* is the broader, general environment (balanced versus biased); *Rew* is reward magnitude; *Speed* is dot speed; ITI is inter-trial interval; *PastRew* is the reward obtained on the preceding trial; *PastactTime* is the observed *actTime* on the preceding trial. Error bars are standard error of the estimated coefficients.

(H) Monkeys waited longer before responding on "medium offer" trials in the biased compared with balanced environment, where the value of the "medium offer" was worth less than the average value of the environment. HRV, higher relative value; LRV, low relative value.

(I) There was no significant difference between the missed "medium offer" trials between the two environments. In (H) and (I), the gray columns are the mean across animals, error bars are the SEM across animals, and each line is data from individual animals. *p < 0.05.

CellPress
OPEN ACCESS

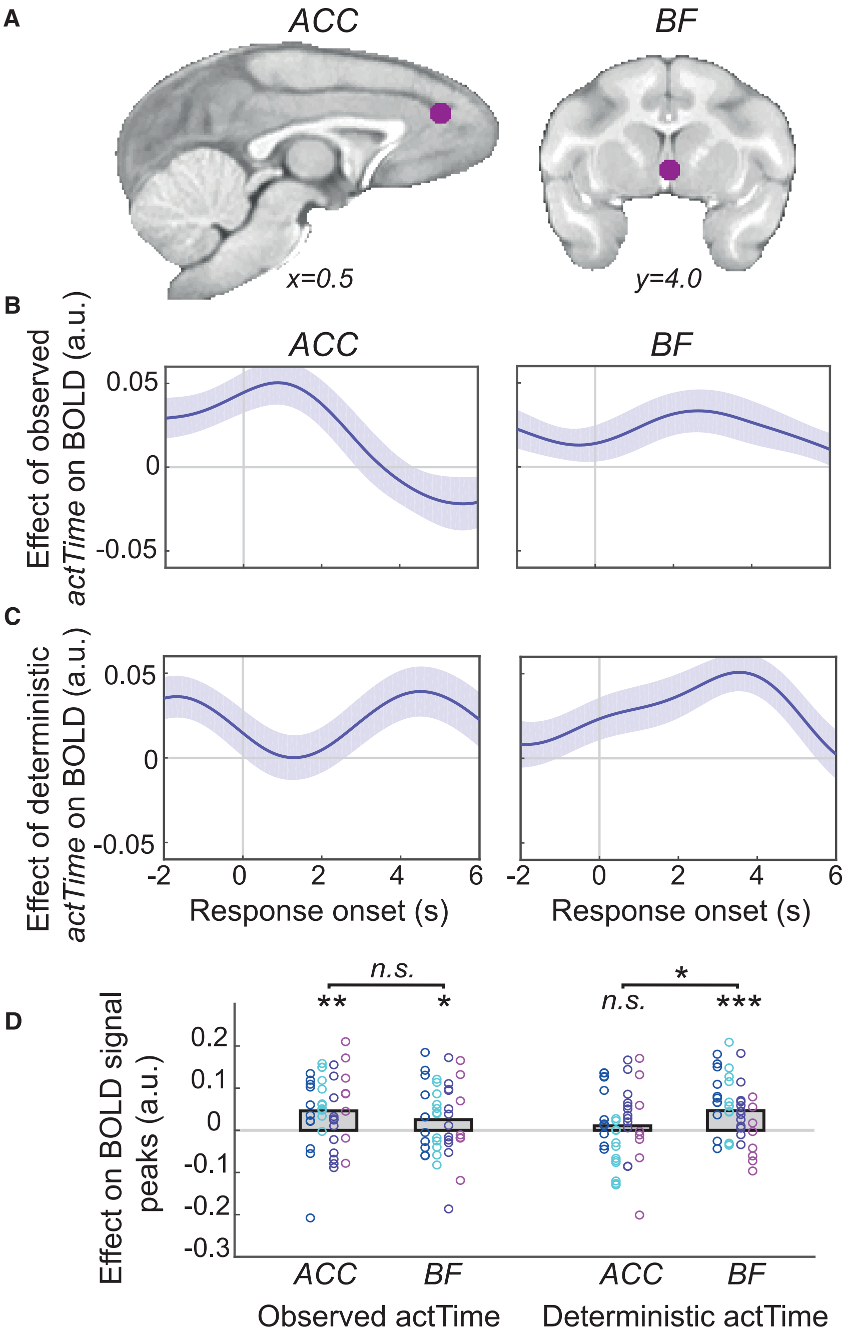

Figure 2. Basal forebrain mediates the influence of the immediate context on animals' time to act

(A) ACC and BF masks were reproduced from Khalighinejad et al.[7] The BF mask contains parts of medial septum/diagonal band of Broca.

(B) The effect of observed *actTime* on BOLD activity in ACC and BF (GLM2.1).

(C) The effect of deterministic *actTime* on BOLD activity in ACC and BF (GLM2.2). The lines and shadings show mean and standard error of β weights across scanning sessions (n = 43), respectively. Time zero is the response time.

(D) Significance testing on time course data was performed by using a leave-one-out procedure on the group peak signal (STAR Methods). Each color represents one animal, and each ring is the peak β weight of one scanning session. The gray columns illustrate the group mean. One-sample and paired-sample t tests. *p < 0.05, **p < 0.01, ***p < 0.001.

replicate these results. We extracted and averaged BOLD signals from voxels within spherical masks centered on the peak of previously observed activation in ACC and BF (STAR Methods; Figure 2A). Activity in both the ACC and BF was significantly correlated with parametric variation in observed *actTime* (leave-one-out test on group peak signals [n = 43]; ACC, $t(42)$ = 3.55, p = 0.001, d = 0.54; BF, $t(42)$ = 2.02, p = 0.049, d = 0.31; GLM2.1; STAR Methods; Figures 2B and 2D). Next, we used a Cox regression model to estimate the time at which an animal is predicted to make a response given the influence of the present and recent past context (STAR Methods). This so-called deterministic *actTime* reflects the proportion of variance in the "observed" *actTime* explained by immediate context. BF ($t(42)$ = 4.19, p < 0.001, d = 0.64), but not ACC ($t(42)$ = 0.80, p = 0.43), integrated features of the immediate context to construct the deterministic component of *actTime* on a trial-wise basis. Importantly, the relationship between BOLD and deterministic *actTime* was stronger in BF than ACC, validating previous findings[7] ($t(42)$ = 2.44, p = 0.02, d = 0.37; GLM2.2; STAR Methods; Figures 2C and 2D).

It has been suggested that DRN tracks the average reward rate in an environment.[8] Here, we showed that the average value of the environment influences animals' observed *actTime* (Figure 1H). Therefore, we asked whether this effect is mediated by DRN. To answer this question, we extracted and averaged the BOLD time course of each voxel within an anatomical mask covering DRN (STAR Methods; Figure 3A). The resulting DRN time course was then compared across "medium offer" trials between the two environments (balanced versus biased design; GLM2.3; STAR Methods; Figure 1E). We found a significant main effect of "broader, general environment" on DRN BOLD activity (leave-one-out test on group peak signals across animals [n = 4]; $t(3)$ = 10.16, p = 0.002, d = 5): across "medium offer" trials, DRN was more active when the value of the offer was worth less than the average value of the environment (i.e., low relative value) compared with higher relative value trials (Figures 3B and 3F). Note that the model contained observed *actTime* as a covariate; therefore, the effect of the "broader, general environment" could not be explained by the difference in *actTime* alone (GLM2.3; main effect of observed *actTime* [p = 0.12]; interaction

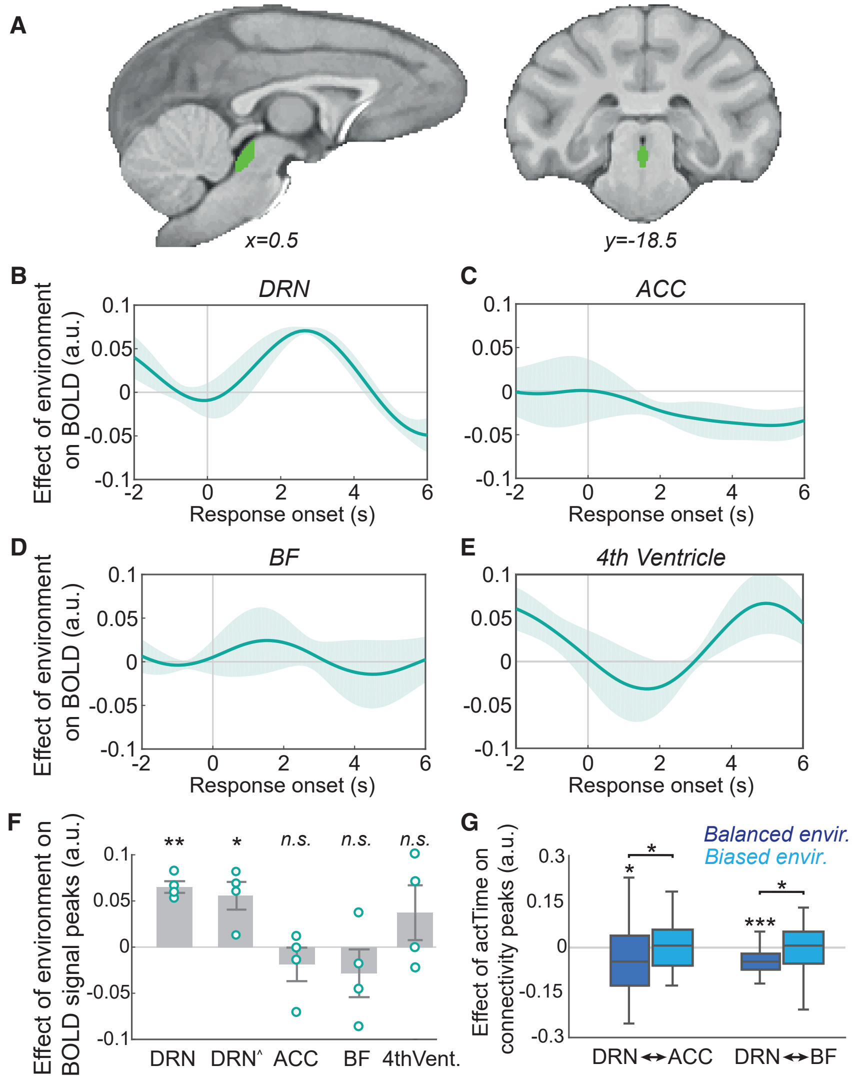

Figure 3. Dorsal raphe nucleus mediates the influence of the broader, general features of the environment on animals' time to act

(A) The DRN anatomical mask (STAR Methods).

(B–E) The effect of environment on BOLD activity across "medium offer" trials—low relative value (Experiment 1) versus higher relative value[7] trials (LRV > HRV)—extracted from DRN (B), ACC (C), BF (D), and the fourth ventricle (E) masks. The lines and shadings show mean and standard error of the β weights across animals (n = 4), respectively. Time zero is the response time.

(F) Significance testing on time course data was performed by using a leave-one-out procedure on the group peak signal (STAR Methods). DRN^ is the effect of environment on DRN BOLD when including the time course from the 4th ventricle, and its interaction with the environment, as confound variables in GLM2.3. The gray columns are mean peak β weights across animals, error bars are SEM across animals, and each ring indicates peak β weight from individual animals.

(G) Significance testing on connectivity data was performed by using a leave-one-out procedure on the peak β weights from PPI analyses between DRN-ACC and between DRN-BF with the observed *actTime* as the moderator (Figure S2 illustrates effective connectivity analysis). In box plots, the central line indicates the median and the bottom and top edges of the box indicate the 25th and 75th percentiles, respectively. Whiskers extend to the most extreme data points not considered outliers. Wilcoxon signed-rank test and one-sample t tests. *p < 0.05, **p < 0.01, ***p < 0.001.

effect between the environment and the observed *actTime* [p = 0.42]).

The data from the balanced and biased designs were obtained from the same animals but in different sessions because we are interested in the impact of variation in the "broader, general environment." Thus, it is possible that the observed effect in the DRN is due to some unspecific difference between the two experiments. However, this is unlikely because of the following: (1) the effect of the "broader, general environment" was specific to DRN and not observed in other areas encoding *actTime*, including ACC (p = 0.38) and BF (p = 0.35) (Figures 3C, 3D, and 3F). Importantly, the effect of the "broader, general environment" was significantly varied with the brain region of interest (ROI) (F(2,6) = 5.2, p = 0.049, η_p^2 = 0.63): it was stronger in DRN compared with BF (t(3) = 3.23, p = 0.048) or ACC (t(3) = 4.21, p = 0.024). (2) The effect was limited to "medium" offer trials and was not observed when running the same analysis across all trials (p = 0.17) or when evaluating the effect of immediate recent past and present contextual factors, including the current offer value (all p > 0.07). One potential concern is that DRN's small size and proximity to the fourth ventricle makes it susceptible to artifacts. Therefore, we extracted and averaged BOLD signals from voxels within a mask covering the fourth ventricle, from both the biased and balanced design datasets (STAR Methods). We then used, first, the extracted time course of activity from the ventricle and, second, the time course of activity from the ventricle in interaction with the "broader, general environment" as confound variables in GLM2.3. The result shows that the relationship between the environment and DRN BOLD remains significant even after accounting for a potential difference in unaccounted artifacts between the two experiments (t(3) = 3.72, p = 0.03, d = 1.8; Figures 3E and 3F).

Thus far, we have shown that ACC and BF tracked trial-by-trial variation in *actTime*, but they did not track the "broader, general environment" in a simple or direct way. BF, specifically, influenced *actTime* by integrating "immediate context and consequences" of a trial (Figure 2). On the other hand, DRN activity was correlated with the "broader, general features" of the environment but not the *actTime* and/or the "immediate context and consequences" of a trial. This means that although DRN activity reflected aspects of the environment that affected animals patience/speed of responding, it did not directly encode patience/speed of responding per se (see also Figure 7). This observation raised the possibility that the DRN is functionally connected with ACC and/or BF so

that the different types of influence associated with "immediate context" and "broader, general environment" can both influence *actTime*. If this is the case, then we should expect that not only will we find evidence of activity coupling between the areas but that such coupling should depend on *actTime* and average value of the environment. To test this hypothesis, we first performed a psychophysiological interaction (PPI) analysis to estimate *actTime*-dependent changes in functional coupling between DRN-ACC and between DRN-BF, within each environment and across all trials (STAR Methods; GLM2.4; Figure 3G). In the balanced environment there was a significant, *actTime*-dependent, negative relationship between BOLD activity in DRN and ACC (leave-one-out test on group peak signals [n = 45]; $t(44) = -2.60$, $p = 0.013$, $d = 0.39$) and between DRN and BF ($t(44) = -4.26$, $p < 0.001$, $d = 0.63$). No such relationship was found in the biased environment (n = 43; DRN and ACC, $p = 0.50$; DRN and BF, $p = 0.70$). Finally, we compared the strength of these relationships between the two environments. The DRN's *actTime*-dependent coupling with ACC and BF was significantly stronger in the balanced compared with the biased environment (Wilcoxon signed-rank test; DRN and ACC, $Z = -1.97$, $p = 0.049$; DRN and BF, $Z = -2.56$, $p = 0.01$; Figure 3G). Given the time series analysis in the previous section, which showed stronger activity in DRN in the low relative value trials (biased design) compared with higher relative value trials (balanced design), the negative direction of the PPI effect in the balanced environment is consistent with an inhibitory influence between ACC/BF and DRN during which animals acted more rapidly (Figure 1H; also see Figure S2). Overall, these results suggest a complementary role of BF and DRN—in communication with ACC—in regulating decision time to act by mediating the influence of "immediate context" and the "broader, general environment," respectively.

Pharmacological manipulation of the serotonergic system prolongs time to act as a function of the average reward rate of the environment

Previous research has shown that optogenetic activation of DRN 5-HT enhances persistence for future reward particularly when rodents should infer the general features of the environment. For example, with a slowly depleting resource[9] or in blocks of trials with high reward probability and high reward-timing uncertainty.[10] Here, we similarly showed that monkeys' action timing is influenced by the general features of the environment: they waited longer before making a response for potential reward when the value of the offer was less than the average value of the environment (Figure 1H). We also showed that this effect was associated with an increased DRN BOLD activity (Figure 3B) and its interactions with other brain areas, such as ACC and BF (Figure 3G). Based on these observations we predicted the following: (1) increasing the systemic 5-HT levels will prolong the length of time monkeys will wait before initiating a response, and (2) this effect will be more profound when the value of the offer is less than the average value of the environment.

To test these predictions, in a within-subject, placebo-controlled, double-blind, cross-over study (Experiment 2), the serotonergic system was manipulated by protracted oral administration of citalopram—a selective 5-HT reuptake inhibitor commonly used to treat depression (see STAR Methods for details and Table S1 for the testing schedule and the measurement of 5-HT levels in blood). First, we used a mixed-effect model to test whether manipulation of the serotonergic system influences the observed *actTime* (STAR Methods; GLM3.1). The result supported our hypothesis: increasing systemic 5-HT levels significantly prolonged the observed *actTime* (mixed-effect model; $\beta = 0.64$, $\chi^2(1) = 7.69$, $p = 0.005$; Figures 4A and S5 for effect on accumulated reward). There was no difference, however, in the proportion of *actTime* that could only be explained by the combined effect of the "immediate recent past and present context" (i.e., "deterministic" *actTime*; $\chi^2(1) = 0.17$, $p = 0.67$; STAR Methods; GLM3.2; Figures 4B and S3). In summary, in Experiment 1 we showed that increasing the average value of the "broader, general environment," so that the relative value of a medium offer was lower than the average value of the environment, resulted in increased DRN activity and slow responding. Now here, in Experiment 2, we have shown that increasing 5-HT, an important DRN output, similarly causes slower responding.

Next, we sought to investigate further ways in which the effect of 5-HT on observed *actTime* might be related to the "broader, general environment." To test the effect of the "broader, general environment" "within" experiment we exploited the fact that the ITI changes in blocks of 30 trials. This means that average reward rate in a long ITI block is lower than a block with short ITI (Figure S3 for further discussion). Therefore, we predicted that citalopram administration has stronger effects on observed *actTime* during long ITI blocks—where reward rate is low—compared with short ITI blocks. This prediction was indeed supported by a significant interaction effect between drug administration (treatment versus control) and ITI ($\beta = 0.14$, $\chi^2(1) = 4.74$, $p = 0.029$; STAR Methods; GLM3.1; Figure 4C). There was no interaction effect between drug administration and other contextual factors; except for a weak interaction effect with the observed *actTime* on the preceding trial ($\beta = -0.15$, $\chi^2(1) = 3.90$, $p = 0.048$; Figure 4D). This indicates that increasing systemic 5-HT levels enhanced animals' ability to wait before making a response, but this effect was greater in blocks with longer ITI where good opportunities were sparser than their average distribution in the environment. This is in line with the finding from Experiment 1 wherein *actTime* was longer when the value of the offer was less than the average value of the environment.

Next, we checked whether this effect could be explained by other aspects of the behavior, such as animals' overall engagement with the task. We compared the number of missed trials between the treatment and control groups and found no significant difference ($\chi^2(1) = 0.004$, $p = 0.95$; Figure 4E). Finally, we speculated that if the observed effect is directly related to the administered drug, we might expect to find a dose-response effect: the effect of drug should be stronger with higher doses. This was indeed the case: citalopram enhanced the ability to wait before making a response when animals were on the maintenance dose but not during early stages of the experiment when the dose was being built up (build-up treatment versus control, $\chi^2(1) = 1.89$, $p = 0.17$; maintenance treatment versus control, $\beta = 0.48$, $\chi^2(1) = 3.86$, $p = 0.049$; Figure 4F).

BOLD activity in DRN is correlated with inter-trial interval

Experiment 1 showed that monkeys waited longer before making a response when the value of the offer was less than the

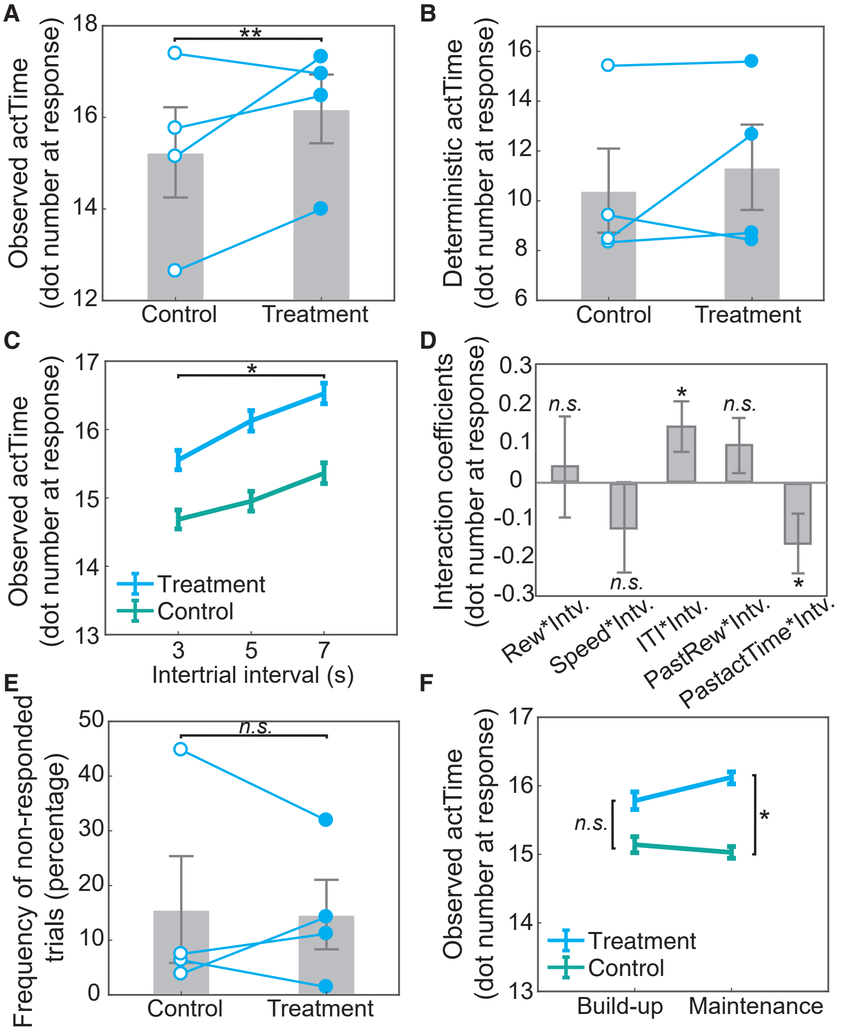

Figure 4. Pharmacological manipulation of the serotonergic system prolongs time to act as a function of the average reward rate of the environment

(A–C) The effect of citalopram (treatment) and placebo (control) on observed *actTime*. Increasing systemic serotonin levels promoted waiting before responding (A). This effect, however, was not observed when using the combined effect of the immediate contextual factors to predict time to act (*deterministic actTime*; B) but showed a significant interaction with ITI, such that the effect of citalopram on observed *actTime* was stronger during long ITI compared with short ITI blocks (C).

(D) Interaction coefficients between drug administration (treatment versus control) and contextual factors in the present and recent past trials.

(E) There was no significant difference in the percentage of non-responded trails between treatment and control conditions.

(F) The effect of citalopram on observed *actTime* was sensitive to the dose of the administered drug. *Intv.* is the drug administration group (treatment versus control). In (A), (B), and (E), the gray column is the mean across animals, error bars are the SEM across animals, and each line is data from individual animals. In (C) and (F), error bars show SEM across data. In (D), error bars are standard error of the estimated coefficients. *$p < 0.05$, **$p < 0.01$. Figure S3 and Table S1 illustrate Cox regression coefficients, the dosing schedule, and effect of drug on 5-HT levels in blood. See also Figure S5.

average value of the environment and that this effect was associated with increased DRN BOLD activity. In a follow-up psychopharmacological study (Experiment 2), we showed that increasing the systemic 5-HT levels prolonged *actTime* and that this effect was greater in blocks with longer ITI, where good opportunities were sparser than their average distribution in the environment. Therefore, if the observed interaction of 5-HT with *actTime* and ITI is driven by a difference in average value of the environment, one might expect the DRN BOLD signal could track ITI.

To test this prediction, we pooled data from both the "balanced" and "biased" designs (88 sessions in total). This was possible because ITI was varied in a similar way in both designs. Importantly, because the ITI effect on BOLD activity is assessed by combining rather than contrasting data across both biased and balanced sessions, it offers the possibility of a powerful test across a larger volume of interest over an extended subcortical region that includes not just DRN but other nuclei that are the origins of other ascending neuromodulatory systems. We found that ITI had an effect on activity in a circumscribed brainstem region that partially overlapped with the anatomically defined DRN ROI previously examined (Z threshold = 3.1; peak Z = 3.9, Caret-F99 Atlas [F99]: x = 1.0, y = −20.5, z = −8.5; small-volume correction; number of voxels = 109, p = 0.04; GLM4.1; Figures 5A and S6). However, no similar effect was seen in other nuclei from which other ascending neuromodulatory systems project, such as the dopaminergic midbrain (Figures 5A and S6). A second analysis tested whether any similar ITI-related changes in activity occurred elsewhere in the brain (Z threshold = 3.1), even if they did not survive cluster correction. The most prominent region to exhibit a related change in activity was in the hippocampus, which is known to play a role in temporal processing and delay discounting.[11,12] Once again, however, there was no evidence of ITI-related activity changes in other areas of interest, including ACC and BF (Figure 5B). Moreover, although ITI had a significant impact on DRN activity, other task variables did not, even when we considered all 88 sessions across both biased and balanced sessions. Finally, we returned to re-examine the DRN effect illustrated in Figure 3B—the effect, on medium offer trials, of the average value of the environment. We confirmed that this effect emerged in the same manner if we examined activity in the DRN ROI that had been defined anatomically a priori (Figure 3A) or if we considered the DRN ROI defined on a functional basis from the ITI contrast (Figure 5C).

Together, these results demonstrate that long ITI—when good opportunities were sparse—was associated with prolonged

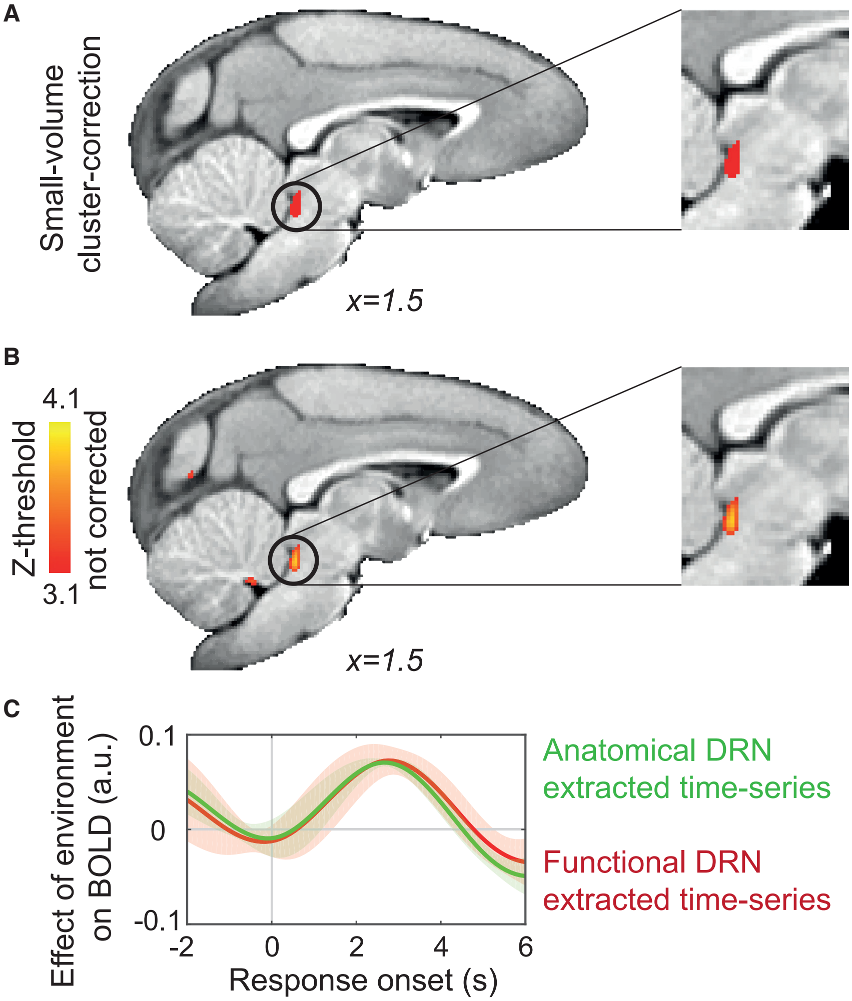

Figure 5. BOLD activity in dorsal raphe nucleus is correlated with inter-trial interval

(A) To demonstrate that within the midbrain and brainstem the effect of ITI on BOLD is limited to DRN and could not be detected at dopaminergic structures, which may encode other aspects of reward rate, we performed a small-volume correction. The DRN cluster emerged as the only significant cluster (number of voxels = 109; p = 0.04).

(B) We also searched for voxels across the whole brain that showed a significant positive correlation between BOLD and ITI (Z > 3.1) even without performing any cluster correction procedure. Clusters were apparent in hippocampus and caudal DRN.

(C) We replicated the effect of the "broader, general environment" (Figure 3B), which was identified in *a priori*-defined anatomical DRN mask (Figure 3A), within the functionally defined DRN cluster identified with ITI contrast (A).

See also Figure S6.

actTime (Figure S1A) and increased activity at DRN. This effect is due to a difference in average value and not simply due to a difference in waiting time between long and short ITIs. This is because Experiment 1 showed that DRN activity was not directly correlated with *actTime*. Nor was there any significant correlation between DRN and *actTime* at the whole-brain level when pooling data from both the "balanced" and "biased" designs (GLM4.1; main effect of observed *actTime*). These results, together with findings from Experiment 1 (Figures 1H and 3B), suggest that the impact of ITI on DRN activity is also likely to be driven by a difference in the average value of the environment. However, whereas in Experiment 1 the effect was observed "between" experiments, we found here a comparable effect "within" experiments. This provides further reassurance that some unspecified difference between biased and balanced sessions had not driven DRN activity changes and action timing changes.

Pharmacological manipulation of the cholinergic system reduces time to act as a function of the immediate recent past and present context

So far, Experiment 1 suggested that DRN influenced *actTime* by tracking the "broader, general environment," while BF influenced *actTime* by integrating features defining the "immediate recent past and present context" of the trial. Experiment 2 showed that pharmacological manipulation of the serotonergic system influenced the relationship between *actTime* and "broader, general environment." In a final experiment (Experiment 3), we asked whether manipulation of the cholinergic system influences the relationship between *actTime* and the "immediate recent past and present context."

To answer this question, in a within-subject, placebo-controlled, double-blind, cross-over study the cholinergic system was manipulated by protracted oral administration of rivastigmine—a cholinesterase inhibitor, which is widely used for the treatment of cognitive deficits in Parkinson's disease (see STAR Methods for details and Table S2 for the testing schedule). We first asked whether manipulating the cholinergic system could influence the observed *actTime*. The length of observed *actTime* was shortened in the treatment compared with the control group (Figure 6A). This effect, however, was not significant at the population level (mixed-effect model; STAR Methods; GLM3.1; $\chi^2(1) = 0.06$, p = 0.80; see also Figure S5 for effect on accumulated reward). Nor was there any significant interaction between drug administration and any particular feature from the immediate recent past and present context (all p > 0.08). This suggests that the effect of ACh on observed *actTime* was not mediated by ITI or any single contextual factor. However, Experiment 1 showed that BF BOLD activity was specifically correlated with the proportion of variance in the observed *actTime* that could be explained by the combined effect of the features in immediate recent past and present context. This "deterministic *actTime*" was therefore estimated for each animal and each trial using a Cox regression model (STAR Methods). We found that the length of time each animal was expected to wait on each trial before making a response, as

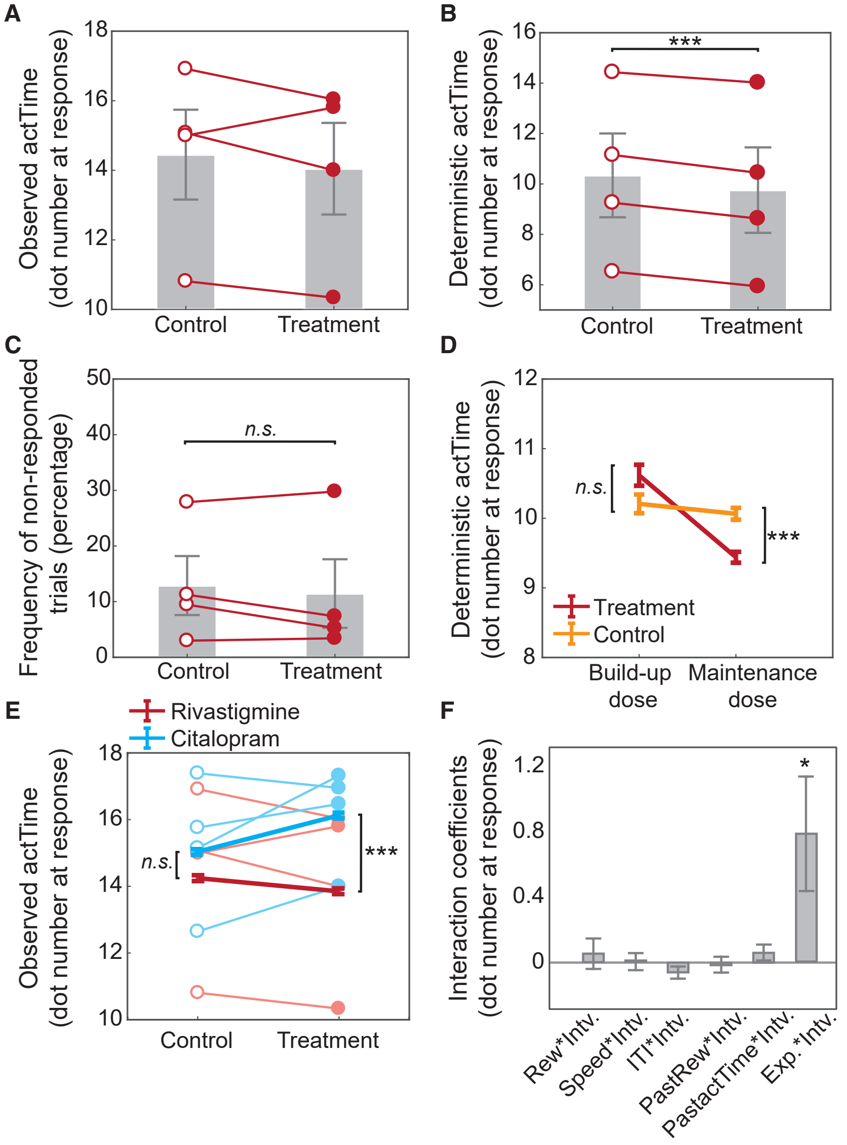

Figure 6. Pharmacological manipulation of the cholinergic system reduces time to act as a function of the immediate recent past and present context

(A–C) The effect of administration of rivastigmine (treatment) and placebo (control) on the observed *actTime* (A), deterministic *actTime* (B), and percentage of non-responded trials (C). (A) Increasing systemic ACh levels reduced observed *actTime* on average, but the effect was not significant at the population level. (B) Increasing ACh levels, however, significantly shortened the “deterministic” component of the *actTime* across all animals.

(C and D) This effect was not due to a change in animals’ overall engagement with the task (C) and was sensitive to the dose of the administered drug (D). In (A)–(C), the gray column is the mean across animals (n = 4), error bars are SEM across animals, and each line is data from individual animals. In (D), error bars show SEM across data.

(E) By pooling data from both Experiments 2 (Figure 4) and 3 (shown here), we found a significant interaction between experiment (*Exp*; rivastigmine versus citalopram) and intervention (*Intv*; treatment versus control).

(F) The interaction coefficients were extracted from GLM3.1 after combining data from both experiments. In (E), thick lines are the mean across data, error bars are the SEM across data, and each line is data from individual animals. In (F), error bars are standard error of the estimated coefficients. *$p < 0.05$, ***$p < 0.001$. See Figure S4 for the Cox regression coefficients and Table S2 for dosing schedule. See also Figure S5.

predicted from their immediate recent past and present context (i.e., *deterministic actTime*), was shortened in the treatment compared with the control group (see Figure S4 for the Cox coefficients for each contextual factor). This effect was significant at the population level (mixed-effect model; STAR Methods; GLM3.2; $\beta = -0.74$, $\chi^2(1) = 43.76$, $p < 0.001$; Figure 6B).

To ensure this effect could not be explained by other aspects of the behavior, such as animals’ overall engagement with the task, we compared the number of trials on which they did not make a response between treatments. There was no significant difference ($\chi^2(1) = 1.67$, $p = 0.20$; Figure 6C). We then asked whether there was a dose-response effect as in Experiment 2: if the observed effect is directly related to the administered drug, we might expect to find a stronger effect with higher doses. There was a significant interaction between treatment group and drug dose (mixed-effect model; STAR Methods; GLM3.3; $\beta = -0.43$, $\chi^2(1) = 5.45$, $p = 0.019$): administration of a cholinesterase inhibitor influenced the combined effect of “immediate context” on animals’ *actTime* but only after the dose was gradually built up and animals reached the maintenance dose (build-up treatment versus control, $\chi^2(1) = 0.58$, $p = 0.45$; maintenance treatment versus control, $\beta = -0.82$, $\chi^2(1) = 48.78$, $p < 0.001$; Figure 6D).

Finally, we designed a grand model comprising data from both Experiment 2 and Experiment 3 to examine whether there was an interaction effect between the experiment (rivastigmine versus citalopram) and drug intervention groups (treatment versus control) on the observed *actTime* (“experiment” × “intervention” fixed and random effects were added to GLM.3.1; STAR Methods). This interaction effect was significant (mixed-effect model; $\beta = 0.76$, $\chi^2(1) = 4.98$, $p = 0.026$; Figures 6E and 6F). Follow-up tests showed that, although administration of rivastigmine shortened *actTime*, citalopram prolonged it ($\beta = 2.36$, $\chi^2(1) = 25.75$, $p < 0.001$). This was not the case when animals were receiving placebo ($\chi^2(1) = 1.20$, $p = 0.27$), suggesting a complementary role of 5-HT and ACh in regulating decision time to act.

DISCUSSION

To decide when to make an action one needs to integrate information about the “immediate context and consequences” of the

action, which may be directly cued by stimuli (as here), and information relating to the "broader, general environment," which may not be immediately observable but only inferable over a longer timescale. Here, animals waited longer before making a response when the value of an identical offer was lower than the average value of the environment (Figure 1H; also see Figure S5 for theoretical accounts of action timing).

The brain activity of the animals was recorded with fMRI while they were performing the task. We focused on predetermined regions of interest, including ACC, BF—containing the medial septum/diagonal band of Broca—and DRN. ACC and BF tracked trial-by-trial variation in observed *actTime*. BF, in particular, encoded the proportion of variance in *actTime* explained by the combined effect of immediate context (i.e., "deterministic" *actTime*)—in line with previous results[7] (Figure 2). DRN, on the other hand, encoded the discrepancy between the value of the current opportunity and the average value of the environment: DRN was more active when the value of the offer was worth less than the average value of the environment (Figure 3). Finally, we found functional coupling between DRN and ACC. Interestingly, strength of coupling depended on the average value of the environment. Interactions between the serotonergic system and other frontal cortical areas have been identified as important in regulating distinct aspects of arousal and learning.[13–16] Here, we show that interactions between DRN and an ACC region near the rostral tip of the cingulate sulcus are important in determining action timing as a function of the richness of the environment (Figure 3).

Activity in DRN was also apparent using a second analysis approach (Figure 5) that focused on ITI. Decision onset-related DRN activity was stronger in task periods in which ITIs were longer and therefore in periods in which reward rate was lower than the average reward rate elsewhere in the same day's testing session. Thus, this effect resembles other decision-related DRN activity changes in this study. A recent fMRI study in NHPs has shown DRN activity encoding global reward state—the amount of reward received regardless of which specific choice is made—in a choice learning task.[8] Although the analysis performed by Wittmann and colleagues was different in important ways from the one reported here, all three analysis approaches, the two used here and the approach taken by Wittmann et al. converge in suggesting that DRN identifies periods in which current opportunities are at odds with those generally available in the environment. The precise details of activity change, including its sign, may depend on the precise time at which activity is recorded (at decision or outcome) and may be clarified further with higher temporal resolution techniques, such as single-neuron recording. Neurophysiological recording has shown that tonic changes in DRN activity occur in relation to expectation of future rewards, including when monkeys are in task blocks in which they might receive either appetitive reward or aversive airpuffs.[17–19] In the former case they also respond to reward delivery or absence on any given trial.

DRN is a small subcortical structure and therefore difficult to image. However, we took multiple approaches to ensure that the reported effect from DRN is not merely artifactual: (1) motion artifacts were carefully cleaned from the raw neuroimaging data. (2) The reported effect was specific to DRN and not observed elsewhere (Figure 3). (3) The DRN effect was specific to the predictor of interest (i.e., average value of environment) and could not be detected when regressing other variables against DRN BOLD. (4) When drawing on data obtained in both balanced and biased sessions, we showed that ITI effects emerged in a region overlapping with the anatomically defined DRN ROI but that similar effects were not seen elsewhere in the brainstem (Figure 5). Future studies with combined fMRI and electrophysiological recording would be useful to further validate the link between fMRI BOLD measurements and DRN neural activity measurements.

DRN provides the majority of serotonergic projections to frontal cortex. Therefore, in a second experiment, we investigated the serotonergic influence on decisions about when to act and its relationship with the average value of the environment. We showed that increasing systemic 5-HT levels by protracted administration of an SSRI prolonged the time animals waited before responding. Administration of SSRIs decreases impulsive behavior in animals.[20,21] This effect, however, is context dependent.[22] In addition, activation of DRN 5-HT neurons promotes waiting for future reward.[23–25] This may reflect enhanced active persistence rather than passive behavioral inhibition.[9] We also showed that 5-HT effects on observed *actTime* were influenced by ITI: the increase in *actTime* was more pronounced during long compared with short ITI blocks. Good opportunities were sparser in long ITI blocks than their average distribution in the environment. This is consistent with data from Experiment 1 where *actTime* was longer when the value of an identical offer was lower than the average value of the environment. A recent study reported that optogenetic stimulation of DRN 5-HT neurons influences learning rate in a decision-making task but only after long ITIs.[26] Although the previous observation concerned learning rate, its dependence on ITI has a clear resemblance to our finding. Finally, we drew a potential link between manipulation of the serotonergic system and DRN BOLD by showing the following: (1) 5-HT prolongs *actTime* but more so during long compared with short ITIs, and (2) ITI is positively correlated with DRN BOLD (Figure 5).

In the final experiment, to investigate the cholinergic role on action timing we used a protracted cholinesterase inhibitor dosing schedule to increase systemic ACh levels. The decision to manipulate the cholinergic system was based on our neuroimaging data, and previous studies in which BF—a cholinergic hub—was identified as determining action timing by mediating the influence of contextual factors in animals' and humans' immediate environments.[7,27] ACh is also linked to cognitive processes, including attention and memory,[28,29] signaling transition in movement state, and invigorating volitional movements.[30–33] Despite the diverse functions of the cholinergic system, a common theme is potentiating action in response to environmental stimuli.[29,34] This is supported by emerging evidence that ACh can rapidly and selectively modulate activity in specific brain areas.[31,33,35] Here, we showed that increasing systemic ACh invigorated movements so that animals acted faster when offered a reward as compared with the control condition. Specifically, it influenced the proportion of variance in action time that could be predicted from immediate stimulus-based contextual information. Together, these results suggest that the cholinergic system influences action timing by employing immediate stimulus-based contextual information, as compared with the broader, general environment. This observation is consistent

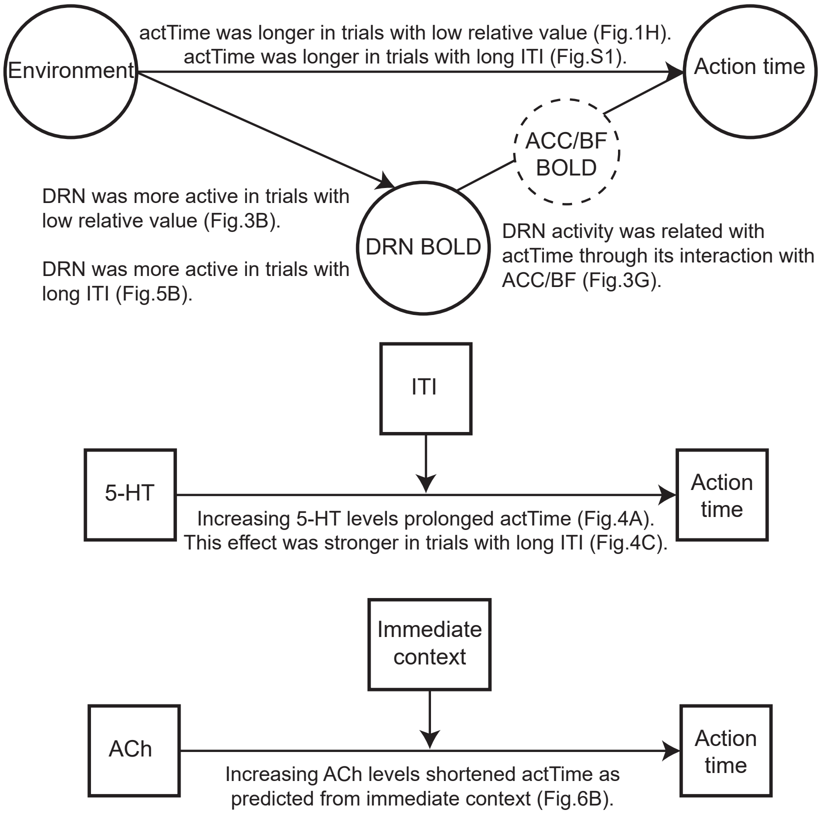

Figure 7. Summary of the results

BF/DRN and the cholinergic/serotonergic systems are involved in modulating decisions about when to act by mediating the influence of specific aspects of the immediate context or wider environment on behavior.

with the role of ACh in invigorating volitional movements in response to environmental stimuli, and the observation that patients with Parkinson's disease often demonstrate degeneration in cholinergic nuclei.[28] Interestingly, rivastigmine has been shown to alleviate the symptoms of apathy in dementia and depression-free patients with Parkinson's disease.[36]

Our study has some limitations: first, unlike in some studies that record from individual neurons, it is not possible to be certain of the identities of neurons contributing to the BOLD signal in the BF and DRN. Second, we manipulated the systemic levels of ACh/5-HT but did not provide direct evidence of increasing ACh/5-HT levels in the macaque brain due to the invasiveness of the necessary procedures. Third, some of the p values were close to the inference cutoff, which warrants future replication studies. Nevertheless, taken together, the results of the current fMRI and pharmacological studies indicate complementary roles for cholinergic and serotonergic systems in decisions about when to act linked to BF and DRN, respectively (Figure 7). These findings may not only help us understand pathological variation in action timing in impulsivity and apathy but also how and why pharmacological interventions that target one or other of these systems might work.

STAR★METHODS

Detailed methods are provided in the online version of this paper and include the following:

- KEY RESOURCES TABLE
- RESOURCE AVAILABILITY
  - Lead contact
  - Materials availability
  - Data and code availability
- EXPERIMENTAL MODEL AND SUBJECT DETAILS
  - Animals
- METHOD DETAILS
  - Experimental task
  - Imaging data acquisition
  - fMRI data preprocessing
  - Pharmacological manipulation
  - Measurement of 5-HT levels in platelet
- QUANTIFICATION AND STATISTICAL ANALYSIS
  - Behavioral analysis
  - Cox regression model
  - ROI-based fMRI data analysis
  - Leave one out on time-series group peak signal
  - Whole-brain fMRI data analysis

SUPPLEMENTAL INFORMATION

Supplemental information can be found online at https://doi.org/10.1016/j.cub.2022.01.042.

ACKNOWLEDGMENTS

This work was funded by the Wellcome Trust (203139/Z/16/Z, WT101092MA, and WT100973AIA) and Medical Research Council (MR/P024955/1). We are very grateful to Dr. Lisa Folkes for performing the blood analyses and to Oxford University Biomedical Services staff for conducting the drug administration and ensuring the welfare of animals. We thank Dr. Miriam Klein-Flügge for commenting on the final version of the manuscript.

CellPress
OPEN ACCESS

AUTHOR CONTRIBUTIONS

N.K. and M.F.S.R. designed the experiment; N.K. collected the data; N.K. analyzed the data; N.K. and M.F.S.R. wrote the manuscript; and S.M. and M.H. advised on pharmacological manipulation and the writing of the manuscript. All authors read and approved the final version of the manuscript.

DECLARATION OF INTERESTS

The authors declare no competing interests.

Received: September 4, 2021
Revised: November 15, 2021
Accepted: January 17, 2022
Published: February 11, 2022

REFERENCES

1. Klaus, A., Alves da Silva, J.A., and Costa, R.M. (2019). What, if, and when to move: basal ganglia circuits and self-paced action initiation. Annu. Rev. Neurosci. *42*, 459–483.
2. Dalley, J.W., and Robbins, T.W. (2017). Fractionating impulsivity: neuropsychiatric implications. Nat. Rev. Neurosci. *18*, 158–171.
3. Husain, M., and Roiser, J.P. (2018). Neuroscience of apathy and anhedonia: a transdiagnostic approach. Nat. Rev. Neurosci. *19*, 470–484.
4. Michelsen, K.A., Schmitz, C., and Steinbusch, H.W.M. (2007). The dorsal raphe nucleus—from silver stainings to a role in depression. Brain Res. Rev. *55*, 329–342.
5. Liu, Z., Lin, R., and Luo, M. (2020). Reward contributions to serotonergic functions. Annu. Rev. Neurosci. *43*, 141–162.
6. Mesulam, M.M., Mufson, E.J., Levey, A.I., and Wainer, B.H. (1983). Cholinergic innervation of cortex by the basal forebrain: cytochemistry and cortical connections of the septal area, diagonal band nuclei, nucleus basalis (substantia innominata), and hypothalamus in the rhesus monkey. J. Comp. Neurol. *214*, 170–197.
7. Khalighinejad, N., Bongioanni, A., Verhagen, L., Folloni, D., Attali, D., Aubry, J.-F., Sallet, J., and Rushworth, M.F.S. (2020). A basal forebrain-cingulate circuit in macaques decides it is time to act. Neuron *105*, 370–384.e8.
8. Wittmann, M.K., Fouragnan, E., Folloni, D., Klein-Flügge, M.C., Chau, B.K.H., Khamassi, M., and Rushworth, M.F.S. (2020). Global reward state affects learning and activity in raphe nucleus and anterior insula in monkeys. Nat. Commun. *11*, 3771.
9. Lottem, E., Banerjee, D., Vertechi, P., Sarra, D., Lohuis, M.O., and Mainen, Z.F. (2018). Activation of serotonin neurons promotes active persistence in a probabilistic foraging task. Nat. Commun. *9*, 1000.
10. Miyazaki, K., Miyazaki, K.W., Yamanaka, A., Tokuda, T., Tanaka, K.F., and Doya, K. (2018). Reward probability and timing uncertainty alter the effect of dorsal raphe serotonin neurons on patience. Nat. Commun. *9*, 2048.
11. Masuda, A., Sano, C., Zhang, Q., Goto, H., McHugh, T.J., Fujisawa, S., and Itohara, S. (2020). The hippocampus encodes delay and value information during delay-discounting decision making. eLife *9*, e52466.
12. Shikano, Y., Ikegaya, Y., and Sasaki, T. (2021). Minute-encoding neurons in hippocampal-striatal circuits. Curr. Biol. *31*, 1438–1449.e6.
13. Alexander, L., Gaskin, P.L.R., Sawiak, S.J., Fryer, T.D., Hong, Y.T., Cockcroft, G.J., Clarke, H.F., and Roberts, A.C. (2019). Fractionating blunted reward processing characteristic of anhedonia by over-activating primate subgenual anterior cingulate cortex. Neuron *101*, 307–320.e6.
14. Barlow, R.L., Alsiö, J., Jupp, B., Rabinovich, R., Shrestha, S., Roberts, A.C., Robbins, T.W., and Dalley, J.W. (2015). Markers of serotonergic function in the orbitofrontal cortex and dorsal raphé nucleus predict individual variation in spatial-discrimination serial reversal learning. Neuropsychopharmacology *40*, 1619–1630.
15. Clarke, H.F., Walker, S.C., Dalley, J.W., Robbins, T.W., and Roberts, A.C. (2007). Cognitive inflexibility after prefrontal serotonin depletion is behaviorally and neurochemically specific. Cereb. Cortex *17*, 18–27.
16. Rygula, R., Clarke, H.F., Cardinal, R.N., Cockcroft, G.J., Xia, J., Dalley, J.W., Robbins, T.W., and Roberts, A.C. (2015). Role of central serotonin in anticipation of rewarding and punishing outcomes: effects of selective amygdala or orbitofrontal 5-HT depletion. Cereb. Cortex *25*, 3064–3076.
17. Bromberg-Martin, E.S., Hikosaka, O., and Nakamura, K. (2010). Coding of task reward value in the dorsal raphe nucleus. J. Neurosci. *30*, 6262–6272.
18. Nakamura, K., Matsumoto, M., and Hikosaka, O. (2008). Reward-dependent modulation of neuronal activity in the primate dorsal raphe nucleus. J. Neurosci. *28*, 5331–5343.
19. Hayashi, K., Nakao, K., and Nakamura, K. (2015). Appetitive and aversive information coding in the primate dorsal raphé nucleus. J. Neurosci. *35*, 6195–6208.
20. Wolff, M.C., and Leander, J.D. (2002). Selective serotonin reuptake inhibitors decrease impulsive behavior as measured by an adjusting delay procedure in the pigeon. Neuropsychopharmacology *27*, 421–429.
21. Costa, V.D., Kakalios, L.C., and Averbeck, B.B. (2016). Blocking serotonin but not dopamine reuptake alters neural processing during perceptual decision making. Behav. Neurosci. *130*, 461–468.
22. Pasquereau, B., Drui, G., Saga, Y., Richard, A., Millot, M., Météreau, E., Sgambato, V., Tobler, P.N., and Tremblay, L. (2021). Selective serotonin reuptake inhibitor treatment retunes emotional valence in primate ventral striatum. Neuropsychopharmacology *46*, 2073–2082.
23. Fonseca, M.S., Murakami, M., and Mainen, Z.F. (2015). Activation of dorsal raphe serotonergic neurons promotes waiting but is not reinforcing. Curr. Biol. *25*, 306–315.
24. Miyazaki, K., Miyazaki, K.W., and Doya, K. (2011). Activation of dorsal raphe serotonin neurons underlies waiting for delayed rewards. J. Neurosci. *31*, 469–479.
25. Miyazaki, K.W., Miyazaki, K., Tanaka, K.F., Yamanaka, A., Takahashi, A., Tabuchi, S., and Doya, K. (2014). Optogenetic activation of dorsal raphe serotonin neurons enhances patience for future rewards. Curr. Biol. *24*, 2033–2040.
26. Iigaya, K., Fonseca, M.S., Murakami, M., Mainen, Z.F., and Dayan, P. (2018). An effect of serotonergic stimulation on learning rates for rewards apparent after long intertrial intervals. Nat. Commun. *9*, 2477.
27. Khalighinejad, N., Priestley, L., Jbabdi, S., and Rushworth, M.F.S. (2020). Human decisions about when to act originate within a basal forebrain–nigral circuit. Proc. Natl. Acad. Sci. USA *117*, 11799–11810.
28. Ballinger, E.C., Ananth, M., Talmage, D.A., and Role, L.W. (2016). Basal forebrain cholinergic circuits and signaling in cognition and cognitive decline. Neuron *91*, 1199–1218.
29. Picciotto, M.R., Higley, M.J., and Mineur, Y.S. (2012). Acetylcholine as a neuromodulator: cholinergic signaling shapes nervous system function and behavior. Neuron *76*, 116–129.
30. Dautan, D., Huerta-Ocampo, I., Gut, N.K., Valencia, M., Kondabolu, K., Kim, Y., Gerdjikov, T.V., and Mena-Segovia, J. (2020). Cholinergic midbrain afferents modulate striatal circuits and shape encoding of action strategies. Nat. Commun. *11*, 1739.
31. Howe, M., Ridouh, I., Allegra Mascaro, A.L., Larios, A., Azcorra, M., and Dombeck, D.A. (2019). Coordination of rapid cholinergic and dopaminergic signaling in striatum during spontaneous movement. eLife *8*, e44903.
32. Jaffe, P.I., and Brainard, M.S. (2020). Acetylcholine acts on songbird premotor circuitry to invigorate vocal output. eLife *9*, e53288.
33. McCormick, D.A., Nestvogel, D.B., and He, B.J. (2020). Neuromodulation of brain state and behavior. Annu. Rev. Neurosci. *43*, 391–415.
34. Záborszky, L., Gombkoto, P., Varsanyi, P., Gielow, M.R., Poe, G., Role, L.W., Ananth, M., Rajebhosale, P., Talmage, D.A., Hasselmo, M.E., et al. (2018). Specific basal forebrain-cortical cholinergic circuits coordinate cognitive operations. J. Neurosci. *38*, 9446–9458.

35. Sarter, M., and Lustig, C. (2020). Forebrain cholinergic signaling: wired and phasic, not tonic, and causing behavior. J. Neurosci. *40*, 712–719.

36. Devos, D., Moreau, C., Maltête, D., Lefaucheur, R., Kreisler, A., Eusebio, A., Defer, G., Ouk, T., Azulay, J.-P., Krystkowiak, P., et al. (2014). Rivastigmine in apathetic but dementia and depression-free patients with Parkinson's disease: a double-blind, placebo-controlled, randomised clinical trial. J. Neurol. Neurosurg. Psychiatry *85*, 668–674.

37. Tustison, N.J., and Avants, B.B. (2013). Explicit B-spline regularization in diffeomorphic image registration. Front. Neuroinform. *7*, 39.

38. Sallet, J., Mars, R.B., Noonan, M.P., Neubert, F.-X., Jbabdi, S., O'Reilly, J.X., Filippini, N., Thomas, A.G., and Rushworth, M.F. (2013). The organization of dorsal frontal cortex in humans and macaques. J. Neurosci. *33*, 12255–12274.

39. Jenkinson, M., Beckmann, C.F., Behrens, T.E.J., Woolrich, M.W., and Smith, S.M. (2012). FSL. NeuroImage *62*, 782–790.

40. Kolster, H., Mandeville, J.B., Arsenault, J.T., Ekstrom, L.B., Wald, L.L., and Vanduffel, W. (2009). Visual field map clusters in macaque extrastriate visual cortex. J. Neurosci. *29*, 7031–7039.

41. Dhurat, R., and Sukesh, M. (2014). Principles and methods of preparation of platelet-rich plasma: a review and author's perspective. J. Cutan. Aesthet. Surg. *7*, 189–197.

42. Bates, D., Maechler, M., Bolker, B., Walker, S., Christensen, R.H.B., Singmann, H., Dai, B., Scheipl, F., Grothendieck, G., Green, P., et al. (2018). lme4: linear mixed-effects models using "Eigen" and S4, https://cran.r-project.org/web/packages/lme4/lme4.pdf.

43. Fox, J., and Weisberg, S. (2018). An R Companion to Applied Regression, Third Edition (SAGE Publications).

44. Murakami, M., Shteingart, H., Loewenstein, Y., and Mainen, Z.F. (2017). Distinct sources of deterministic and stochastic components of action timing decisions in rodent frontal cortex. Neuron *94*, 908–919.e7.

45. Paxinos, G., Huang, X.-F., and Toga, A.W. (1999). The Rhesus Monkey Brain in Stereotaxic Coordinates, First Edition (Academic Press).

CellPress
OPEN ACCESS

STAR★METHODS

KEY RESOURCES TABLE

REAGENT or RESOURCE	SOURCE	IDENTIFIER
Chemicals, peptides, and recombinant proteins		
Isoflurane – ISOFLO 250ml	Centaur	30135687
Ketamine – Narketan 10% 10ml INJ CD(SCH4)1 1-MCD	Centaur	03120257
Midazolam – Hypnoval amps 10mg/2ml	Centaur	23191407
Atropine – Atrocare INJ 25ml	Centaur	02500456
Meloxicam – Metacam INJ 10ml 5mg/ml DOGS/CATS	Centaur	02500456
Ranitidine 50mg/2ml x5 INJ	Centaur	30294115
Saline	DPAG, University of Oxford	N/A
Formalin	DPAG, University of Oxford	N/A
Citalopram 20mg film-coated tablets	Almus Pharmaceuticals	N/A
Rivastigmine 1.5mg capsules	Torrent Pharmaceuticals	N/A
Deposited data		
Behavioral and brain data	This paper	https://github.com/nimakh8/WhenToAct
Experimental models: Organisms/strains		
Macaca mulatta, 4 males, between 4-6 years old, between 11.6-14.2 kg, socially housed	MRC, Centre for Macaques	NCBITaxon:9544
Software and algorithms		
MATLAB 2017a	Mathworks	N/A
Presentation	Neurobehavioral systems	N/A
FMRIB Software Library v5.0	FMRIB, WIN, Oxford, UK	N/A
Advanced Normalization Tools	Tustison and Avants[37]	N/A
Magnetic Resonance Comparative Anatomy Toolbox	Neuroecology Lab	https://github.com/neuroecology/MrCat
Offline_SENSE	Windmiller Kolster Scientific	N/A
R	The R Foundation	N/A
Other		
MRI compatible frame	Crist Instruments	http://www.cristinstrument.com/products/stereotax/stereotax-primate
Four-channel phased-array coil	Windmiller Kolster Scientific	https://www.wkscientific.com/#mri-coils

RESOURCE AVAILABILITY

Lead contact
Further information and requests for resources should be directed to and will be fulfilled by the lead contact, Nima Khalighinejad (nima.khalighinejad@psy.ox.ac.uk)

Materials availability
This study did not generate new unique reagents.

Data and code availability
The behavioral and brain data that support the findings of Exp.1, Exp.2 and Exp.3 are available at: https://github.com/nimakh8/WhenToAct. This paper does not report original code. Any additional information required to reanalyze the data reported in this paper is available from the lead contact upon request.

EXPERIMENTAL MODEL AND SUBJECT DETAILS

Animals

Four male rhesus monkeys (*Macaca mulatta*) were involved in the experiment. They weighed 14.1–16.8 kg and were 6-8 years of age. They were group housed and kept on a 12-hr light dark cycle, with access to water 12–16 hr on testing days and with free water access on non-testing days. All procedures were conducted under licenses from the United Kingdom (UK) Home Office in accordance with the UK The Animals (Scientific Procedures) Act 1986 and with the European Union guidelines (EU Directive 2010/63/EU).

METHOD DETAILS

Experimental task

At the beginning of each trial an empty frame (8 x 26 cm) appeared on the left or right side of the screen. The frame gradually filled with dots (round circles, r = 0.3 cm, max number of dots = 25) emerging from top to bottom (Figure 1B). Animals could terminate the trial, at a time of their own choice, by touching a custom-made infra-red touch sensor, on the side corresponding to the image. The trial continued if they touched the opposite side. The probability of getting reward increased as more dots appeared on the screen, following a sigmoid curve (Figure 1C). The probability distribution was drawn from a sigmoid function. The input to the function was a vector corresponding to the number of dots from 1 to 25. The midpoint of the curve was at dot #12 (50% chance of getting reward) with the steepness of 0.5. The probability distribution was constant across the trials and the sessions. The color of the frame and dots varied from trial to trial but remained constant within a trial. The color indicated potential reward magnitude and could be red, green or blue, indicating one, two or three drops of juice, respectively. In addition to the color, the speed of the dots appearance also varied from trial to trial. A new dot appeared every 100, 200 or 300 ms. The color and the speed of the dots varied independently of one another, and in a pseudo-randomized order. Animals had the option to respond, any time from the beginning of the trial (appearance of the empty frame) to 300 ms after the frame was filled (appearance of the last dot). If they responded, they were offered drops of juice or no juice, based on the probability distribution at the time of response. There was a delay of 4 s between response and outcome (action-outcome delay). Successful (rewarded) and unsuccessful (unrewarded) outcomes were indicated by an upward and downward pointing triangle, respectively. The triangle remained on the screen for 2 s. If rewarded, drops of blackcurrant juice were delivered by a spout placed near the animal's mouth during scanning. Each drop was composed of 1 ml blackcurrant juice. No juice was delivered when the trial was not rewarded. After the outcome phase, they proceeded to the next trial after a 3, 5 or 7 s inter-trial interval (ITI). ITI varied in blocks of 30 trials in a pseudo-randomized order. Specific patterns on the left and right side of the screen indicated the ITI block (Figure 1D). If animals did not respond by 300ms after the emergence of the last dot, the frame disappeared, and they had to wait for 4 s (equivalent to action-outcome delay) + 3, 5 or 7 s (ITI) for the next trial to start. Animals were given 40min to perform the task at each session. The task finished after 40 min, regardless of the number of trials performed.

This original (balanced) design was used for the pharmacological studies (Exp.2&3). However, for the neuroimaging experiment (Exp.1), to investigate the effect of the environment on action time, we manipulated the distribution of the offers. In the 'balanced' design the good (large reward and fast dot speed), medium and bad (small reward and slow dot speed) offers were distributed equally, i.e., there were equal numbers of trials with large, medium and small reward magnitudes and equal number of trials with slow, medium and fast dot speeds. In Experiment 1 (biased design), this distribution was skewed in favor of good offers, i.e., there were more trials with large (46% of the trials) compared to small (21%) reward magnitude and more trials with fast (46%) compared to slow (21%) dot speed. However, importantly, there were equal number of medium offers (medium reward and medium dot speed; 33% of the trials) in both the 'balanced' and the 'biased' design. This enabled us to compare the effect of the environmental context on action time in medium offer trials. The experiment was controlled by Presentation software (Neurobehavioral Systems, Albany, CA).

Imaging data acquisition

Awake-animals (N = 4) were head-fixed in a sphinx position in an MRI-compatible chair (Rogue Research, MTL, CA). MRI was collected using a 3T horizontal bore MRI clinical scanner and a four-channel phased array receive coil in conjunction with a radial transmission coil (Windmiller Kolster Scientific Fresno, CA). Each loop of the coil had an 8cm diameter which ensures a good coverage of the animal's head. The chair was positioned on the sliding bed of the scanner. The receiver coils were placed on the side of the animal's head with the transmitter placed on top. Animals' responses were registered by custom-made MRI-compatible infra-red touch sensors. An MRI-compatible screen (MRC, Cambridge) was placed 30cm in front of the animal and the image was projected on the screen by a LX400 projector (Christie Digital Systems). Functional data were acquired using a gradient-echo T2* echo planar imaging (EPI) sequence with a 1.5 x 1.5 x 1.5 mm resolution, repetition time (TR) 2.28 s, echo time (TE) 30 ms and flip angle 90°. At the end of each session, proton-density-weighted images were acquired using a gradient-refocused echo (GRE) sequence with a 1.5 x 1.5 x 1.5 mm resolution, TR 10 ms, TE 2.52 ms, and flip angle 25°. These images were later used for offline MRI reconstruction. T1-weighted MP-RAGE images with a resolution of 0.5 x 0.5 x 0.5 mm, TR 2.5 s, TE 4.04 ms, inversion pulse time (TI) 1.1 s, and flip angle 8°, were acquired in separate sessions under general anesthesia. Anesthesia was induced by intramuscular injection of 10 mg/kg ketamine, 0.125-0.25 mg/kg xylazine, and 0.1 mg/kg midazolam and maintained with isoflurane.[38] Anesthesia was only used for collecting T1-weighted structural images.

fMRI data preprocessing

Data preprocessing was performed following previously reported methods[7] and using tools from FMRIB Software Library (FSL),[39] Advanced Normalization Tools (ANTs; http://stnava.github.io/ANTs),[37] and the Magnetic Resonance Comparative Anatomy Toolbox (MrCat; https://github.com/neuroecology/MrCat). First, T2* EPI images acquired during task performance were reconstructed by an offline-SENSE method that achieved higher signal-to-noise and lower ghost levels than conventional online reconstruction[40] (Offline_SENSE GUI, Windmiller Kolster Scientific, Fresno, CA). A low-noise EPI reference image was created for each session, to which all volumes were non-linearly registered on a slice-by-slice basis along the phase-encoding direction to correct for time-varying distortions in the main magnetic field due to body and limb motion. The aligned and distortion-corrected functional images were then non-linearly registered to each animal's high-resolution structural images. A group specific template was constructed by registering each animal's structural image to the CARET macaque F99 space.[40] Finally, the functional images were temporally filtered (high-pass temporal filtering, 3-dB cutoff of 100s) and spatially smoothed (Gaussian spatial smoothing, full-width half maximum of 3mm). Three measures were used to detect artefacts in the data: a) For each slice in each volume the linear transform (in the y-plane) from that slice to the corresponding slice in the mean reference image; b) The normalized correlation between that slice and the corresponding slice in the mean reference image; c) For each volume, the correlation between that volume (mean-filtered across z-slices) and the mean reference image after correction. Volumes were removed when they exceeded 2.5 SDs above the median of each measure. The threshold was chosen to keep the number of censored volumes less than 10% of the total volumes. We also added as parametric regressors, 13 PCA components that describe, for each volume, the warping from that volume to the mean reference image when correcting motion artefacts (i.e., they capture signal variability associated with motion induced distortion artefacts), as regressors of non-interest that were not convolved in our general linear models.

Pharmacological manipulation

For Experiment 2 systemic doses of a selective serotonin reuptake inhibitor (Citalopram 20mg tablets) were administered via oral route by mixing the crushed tablet with animals' routine daily food. Four monkeys (same cohort as in Exp.1) were randomly divided into two groups. The treatment group received ½ of the tablet (10mg, once a day) mixed with their food. The control group received their food at the same time without it being mixed with the drug. At the end of the first week the dose was increased to 1 tablet (20mg, once a day). At the end of the second week animals were kept on 20mg/day for another 10 days and were tested on the experimental task on alternate days. Data collected during the last 10 days were used for the main analyses (5 sessions per animal). In both the treatment and control groups, behavioral testing was conducted at the same time of the day, 90min after the afternoon dose. This timing was chosen based on the pharmacokinetic properties of citalopram (in humans, peak plasma concentrations are reached in approximately 2-4 hours). The experimental task was similar to Exp.1 but with a 'balanced' design schedule (i.e., with equal number of good, medium and bad offers). At the end of the 10th day, drug administration was stopped, and monkeys were given a two-week wash-out period. At the end of the wash-out period, the treatment and control groups were switched, and the same protocol was followed (see Table S1 for the testing schedule).

Experiment 3 followed the same protocol as in Experiment 2 but used a different dosing regimen. Systemic doses of a cholinesterase inhibitor (Rivastigmine Sandoz, 1.5mg capsule) were administered via an oral route by mixing the content of the capsule with animals' routine daily food, using a gradually increasing dosing schedule. Four monkeys were randomly divided into two groups (same cohort as in Exp.2). The treatment group received ¼ content of the capsule (~0.37mg, twice a day) mixed with their food. The control group received their food at the same times without it being mixed with the drug. At the end of the first week the dose was increased to ½ capsule (~0.75mg, twice a day). At the end of the second week the treatment group was put on the full dose (1.5mg twice a day). The animals remained on the full dose/placebo for 10 days and were tested on the experimental task on alternate days. Data collected during the last 10 days were used for the main analyses (5 sessions per animal). In both the treatment and control groups, behavioral testing was conducted at the same time of the day, 30min after the afternoon dose. This timing was chosen based on the pharmacokinetic properties of rivastigmine (in humans, peak plasma concentrations are reached in approximately 1 hour). At the end of the 10th day, drug administration was stopped, and monkeys were given a two-week wash-out period. At the end of the wash-out period, the treatment and control groups were switched, and the same protocol was followed (see Table S2 for the testing schedule). Exp.3 was conducted four months after Exp.2, in the same monkeys.

Both experiments (Exp.2&3) had a "within-subject" design: animals acted as their own control at different time points. Importantly, doses were prepared by the facility staff and the experimenter was blind to the type of intervention during the whole data collection process. No adverse effect was observed from dosing in Exp.2 or Exp.3.

Measurement of 5-HT levels in platelet

Blood samples were taken from macaques on the last day of the dosing schedule. Platelet rich plasma (PRP) samples were prepared by following the method from Dhurat and Sukesh.[41] Samples were then frozen for later HPLC analysis. Bovine serum albumin (BSA) and serotonin HCl were obtained from Sigma-Aldrich. Ammonium formate, acetonitrile and formic acid were obtained from Fisher Scientific UK. PBS was from Oxoid, UK. 5% BSA/PBS was used as a surrogate matrix for serotonin analysis. Calibration curves were measured from 0.025 – 5 μmoles/L. No internal standards were used. 50 μl standard or plasma sample were mixed with 250 μl acetonitrile. Samples were vortexed for 10 sec then spun (13000 x g, 5 min, 4 °C) and the supernatant dried down in a heated centrifugal evaporator in brown glass vials. Samples were reconstituted in 50 μl 10 mM ammonium formate pH 3.5, for injection. Separation was achieved using a Waters Acquity UPLC system and a Waters Atlantis T3 column (3 μm, 150 x 3.0 mm) at 35°C, detection

was on a Waters TQD mass spectrometer. Eluents comprised of A: 10 mM ammonium formate pH 3.5; B: acetonitrile with a gradient of 10-90 % B in 5 min with a flow rate of 0.4 ml/min. Serotonin was detected in electrospray positive mode (ES+) with SIR at 176.9 (M+H) (see Table S1 for results).

QUANTIFICATION AND STATISTICAL ANALYSIS

Behavioral analysis

We used linear mixed-effect models (LMEM) to assess the effect of the environmental features and pharmacological manipulation on the observed and deterministic *actTime*. To maintain a type-I error rate of 5%, in addition to the usual 'fixed' effects the LMEMs contained by-subject and by-session random intercepts, and by-subject random slopes. The maximum likelihood method was used for model estimation. The modelling was performed with the 'lme4' package in R.[42] For inferential statistics for a given fixed effect, Wald Chi-square tests were calculated using the Anova function with the 'car' package in R.[43] We used the following models:

GLM1.1

$$\begin{aligned} &observedactTime_t \\ &\quad = \beta_0 + \beta_1 environment_t + \beta_2 magRew_t \\ &\quad + \beta_3 dotSpd_t + \beta_4 ITI_t + \beta_5 rewardOutcome_{t-1} + \beta_6 actTime_{t-1} \\ &\quad + \beta_7 (magRew_t \times environment_t) + \beta_8 (dotSpd_t \times environment_t) \\ &\quad + \beta_9 (ITI_t \times environment_t) + \beta_{10} (rewardOutcome_{t-1} \times environment_t) \\ &\quad + \beta_{11} (actTime_{t-1} \times environment_t) + \mu_0 + \mu_1 environment + \mu_2 magRew_t \\ &\quad + \mu_3 dotSpd_t + \mu_4 ITI_t + \mu_5 rewardOutcome_{t-1} + \mu_6 actTime_{t-1} \\ &\quad + \mu_7 (magRew_t \times environment_t) + \mu_8 (dotSpd_t \times environment_t) \\ &\quad + \mu_9 (ITI_t \times environment_t) + \mu_{10} (rewardOutcome_{t-1} \times environment_t) \\ &\quad + \mu_{11} (actTime_{t-1} \times environment_t) + v_0 + e, \end{aligned}$$

where β_{0-11} are the fixed effects, μ_0 is by-subject random intercept, μ_{1-11} are by-subject random slopes, and v_0 is by-session random intercept. *observed actTime* is the number of dots on the screen at the time of response on trial *t*. *environment* is the biased design vs. the balanced design. *magRew* is the reward magnitude on trial *t*, *dotSpd* is the dot speed on trial *t*, *ITI* is the inter-trial interval on trial *t*, *rewardOutcome* is the obtained reward on trial *t-1*, and *actTime* is the observed action time on trial *t-1*.

GLM1.2

$$\begin{aligned} &observedactTime_{t(m)} \\ &\quad = \beta_0 \\ &\quad + \beta_1 environment_{t(m)} + \beta_2 ITI_{t(m)} + \beta_3 rewardOutcome_{t(m)-1} \\ &\quad + \beta_4 actTime_{t(m)-1} + \beta_5 (environment_{t(m)} \times ITI_{t(m)}) + \beta_6 (environment_{t(m)} \\ &\quad \times rewardOutcome_{t(m)-1}) + \beta_7 (environment_{t(m)} \times actTime_{t(m)-1}) \\ &\quad + \mu_0 + \mu_1 environment_{t(m)} + \mu_2 ITI_{t(m)} + \mu_3 rewardOutcome_{t(m)-1} \\ &\quad + \mu_4 actTime_{t(m)-1} + \mu_5 (environment_{t(m)} \times ITI_{t(m)}) + \mu_6 (environment_{t(m)} \\ &\quad \times rewardOutcome_{t(m)-1}) + \mu_7 (environment_{t(m)} \times actTime_{t(m)-1}) + v_0 + e, \end{aligned}$$

where *m* is a 'medium offer' trial (trials with medium reward magnitude and medium dot speed). This model is similar to GLM1.1 with the difference that *magRew* and *dotSpd* were dropped from the model because they do not vary across 'medium offer' trials.

GLM3.1

$$\begin{aligned} &observedactTime_t \\ &\quad = \beta_0 + \beta_1 intervention + \beta_2 magRew_t \\ &\quad + \beta_3 dotSpd_t + \beta_4 ITI_t + \beta_5 rewardOutcome_{t-1} + \beta_6 actTime_{t-1} \\ &\quad + \beta_7 (magRew_t \times intervention_t) + \beta_8 (dotSpd_t \times intervention_t) \\ &\quad + \beta_9 (ITI_t \times intervention_t) + \beta_{10} (rewardOutcome_{t-1} \times intervention_t) \\ &\quad + \beta_{11} (actTime_{t-1} \times intervention_t) + \mu_0 + \mu_1 intervention + \mu_2 magRew_t \\ &\quad + \mu_3 dotSpd_t + \mu_4 ITI_t + \mu_5 rewardOutcome_{t-1} + \mu_6 actTime_{t-1} \\ &\quad + \mu_7 (magRew_t \times intervention_t) + \mu_8 (dotSpd_t \times intervention_t) \\ &\quad + \mu_9 (ITI_t \times intervention_t) + \mu_{10} (rewardOutcome_{t-1} \times intervention_t) \\ &\quad + \mu_{11} (actTime_{t-1} \times intervention_t) + v_0 + e, \end{aligned}$$

where *Intervention* is the drug manipulation group (treatment vs control group).

GLM3.2

$$deterministicactTime_t = \beta_0 + \beta_1 intervention_t + \mu_0 + \mu_1 intervention_t + v_0 + e,$$

CellPress
OPEN ACCESS

where *deterministic actTime* is number of dots on the screen at which an animal is expected to make a response given the influence of the environment relating to both immediate present context and the immediate recent past context, as measured by the Cox regression model (see below).

GLM3.3

$$observedactTime_t = \beta_0 + \beta_1 intervention_t + \beta_2 dose_t + \beta_3(intervention_t \times dose_t) + \mu_0 + \mu_1 intervention_t + \mu_2 dose_t \quad + \mu_3(intervention_t \times dose_t) + e,$$

where *dose* is the administered dose of the drug (build-up dose vs. maintenance dose). By-session random intercept (v_0) is not included in this model because *dose* fixed-effect (β_2) varies between testing sessions.

Cox regression model

To estimate the deterministic component of action time we used a specific class of survival models called the Cox proportional hazard model. The model predicts time-to-event (*actTime*) on the current trial from present and past contextual factors. Specifically, the predictors (covariates) included reward magnitude, dot speed, and ITI of the current trial, and the actual reward and *actTime* on the past 10 trials. The model is described as:

$$\lambda(t) = \lambda_0(t).\exp(\beta \mathbf{x}),$$

where $\lambda(t)$represents a hazard function (hazard rate of responding), $\lambda_0(t)$ represents a baseline hazard function, that is a hazard function when all the covariates are 0, β is a row vector with 23 elements (3 present contextual factors + 10 past rewards + 10 past *actTimes*) representing Cox coefficients for each covariate and **x** is a 23 element column vector representing covariates, present contextual factors and contextual factors of the past 10 trials. The coefficients were estimated for each testing session by using the 'coxphfit' function in MATLAB.

A detailed method for obtaining Cox coefficients has been previously described.[44] The estimated Cox coefficients $(\widehat{\beta})$ from the predictors on the current trial and the past 10 trials were used to obtain the expected *actTime* by the following method: First, the cumulative hazard function, $\widehat{\Lambda}_x(t)$, of each trial was estimated given the baseline cumulative hazard function, $\widehat{\Lambda}_0(t)$, and the covariates:

$$\widehat{\Lambda}_x(t) = \widehat{\Lambda}_0(t).\exp(\widehat{\beta}\mathbf{x}),$$

The cumulative hazard function of each trial was then used to estimate the survival function of each trial, *S(t)*:

$$\widehat{S}_x(t) = \exp\left(-\widehat{\Lambda}_x(t)\right),$$

The deterministic *actTime* is estimated by:

$$[actTime] = \int_0^\infty \widehat{S}_x\,(t),$$

ROI-based fMRI data analysis

The region of interest (ROI)-based analysis was conducted on fMRI data obtained from four macaques (number of scanning sessions=43; 11 scans per monkey except M1 with 10 scans). The ACC and BF masks were reproduced from a previous study.[7] The anatomical DRN and fourth ventricle masks were designed in the group template F99 space using the Rhesus Monkey Brain Atlas.[45] Masks were then transformed from the standard space to each individual animal functional space by applying a non-linear transformation. For time-series analyses, the filtered time-series of each voxel within the masks were averaged, normalized and up-sampled. The up-sampled data was then epoched in 8 s windows, time-locked to either the trial onset (decision time) or the moment each animal made a response (response time). Time-series GLMs were then fit at each time step of the epoched data, in responded trials, using ordinary least squares (OLS). We ran the following models:

GLM2.1

$$BOLD_t = \beta_0 + \beta_1\ observed\_actTime_t + \beta_2\ time_t + e\ ,$$

where *BOLD* is a *t x i* (*t* trial, *i* time samples) matrix containing the times series data for a given ROI. $\beta_0$is an unmodulated regressor controlling for the constant effects of stimulus presentation and action execution. *time* is a confound regressor representing the time passed since the beginning of the scanning session.

GLM2.2

$$BOLD_t = \beta_0 + \beta_1\ deterministic\_actTime_t + \beta_2\ observed\_actTime_t + \beta_3\ time_t + e\ ,$$

where *deterministic_actTime* is the number of dots at which animals ought to make a response as predicted by the Cox regression model from present and recent past contextual factors.

GLM2.3

$$BOLD_{t(m)} = \beta_0 + \beta_1\, environment_{t(m)} + \beta_2\, observed\_actTime_{t(m)} + \beta_3 \left(environment_{t(m)} \times observed\_actTime_{t(m)}\right) + \beta_4\, time_{t(m)} + e\,,$$

where *m* is a 'medium offer' trial (trials with medium reward magnitude and medium dot speed). *environment* is the biased design vs. the balanced design.

GLM2.4

$$BOLD_t = \beta_0 + \beta_1\, seedBOLD_t + \beta_2\, observed\_actTime_t + \beta_3\, PPI_t + \beta_4\, time_t + e\,,$$

where *BOLD* is BOLD activity at ACC or BF. *seedBOLD* is BOLD activity at DRN. *PPI* is the interaction between *seedBOLD* and *observed_actTime*.

Leave one out on time-series group peak signal

Significance testing on time-course data was performed by using a leave-one-out procedure on the group peak signal to avoid potential temporal selection biases. For every scanning session, we calculated the time course of the group mean beta (β) weights of the relevant regressor based on the remaining sessions (44 scanning sessions in the 'balanced' design[7] and 42 scanning sessions in the 'biased' design). We then identified the (positive or negative) group peak of the regressor of interest within the full width of the epoched time course (8 s windows). Next, we took the beta weight of the remaining session at the time of the group peak. We repeated this for all sessions. Therefore, the resulting peak beta weights (45 peaks in the 'balanced' design and 43 peaks in 'biased' design) were selected independently from the time course of each single session. We assessed significance using two-tailed, one-sample t tests on the resulting beta weights.

The same procedure was followed when comparing BOLD activity in 'medium offer' trials between the two designs. However, rather than calculating beta weights within each scanning session, BOLD signal from 'medium offer' trials were pooled across scanning sessions within each monkey. This was done in this way because of the relatively low number of 'medium offer' trials within each scanning session and in order to produce less noisy estimates of effects. Therefore, the group mean beta weights of the relevant regressor were identified using a leave-one-out procedure on the group peak signal across monkeys (N = 4) rather than scanning sessions.

Whole-brain fMRI data analysis

To investigate the ITI effect we searched for voxels – across the whole-brain – in which BOLD activity was positively correlated with parametric variation in ITI. To perform the whole brain analysis a univariate generalized linear model (GLM) framework was implemented in FSL FEAT. At the first level, a GLM was constructed to compute the parameter estimates (PEs) for each regressor:

GLM4.1

$$\begin{aligned} BOLD = {} & \beta_0 + \beta_1\, resp + \beta_2\, magRew + \beta_3\, dotSpd + \beta_4\, ITI + \beta_5\, pastRewardOutcome \\ & + \beta_6\, pastObserved\_actTime + \beta_7\, Observed\_actTime + \beta_8\, time + \beta_9\, rightconv \\ & + \beta_{10}\, leftconv + \beta_{11}\, mainOut + \beta_{12}\, levelOut + \beta_{13}\, rightunconv + \beta_{14}\, leftunconv \\ & + \beta_{15}\, mouth, \end{aligned}$$

where *BOLD* is a t x 1 (t time samples) column vector containing the times series data for a given voxel. *resp* is an unmodulated regressor representing the main effect of stimulus presentation in responded trials (all event amplitudes set to one). *magRew, dotSpd* and *ITI* are parametric regressors with three levels, which represent reward magnitude, speed of dots, and inter-trial-interval on the current trial, respectively. *pastRewardOutcome* is a parametric regressor with four levels representing the reward outcome on the past trial. *pastObserved_actTime* is also parametric and represents *actTime* on the past trial. *pastRewardOutcome* and *pastObserved_actTime* were both weighted by their influence on *actTime* on the current trial (multiplied by their coefficients from behavioural GLM). Observed_*actTime* represents time-to-act (number of dots at response) on the current trial. Regressors 1 to 7 were all boxcar regressors with a duration of 500 ms that were convolved with a hemodynamic response function (HRF) specific for monkey brains. Regressors 1-6 were all time-locked to the onset of the trial. Regressor 7 started 500 ms before animals made a response by cutting the infra-red touch sensor and continued for 500 ms. Regressors 8-15 were task-related confound regressors. *time* is a parametric regressor representing the time passed since the beginning of the scanning session and is locked to the trial onset. *leftconv* and *rightconv* are unmodulated regressors (all event amplitudes set to one), locked to 500 ms prior to response, representing the response with the left and right hand, respectively. *mainOut* is an unmodulated regressor representing the main effect of outcome (all event amplitudes set to one). *levelOut* is a parametric regressor with four levels representing the reward outcome on the current trial. Regressors 11-12 were locked to the onset of outcome (juice) delivery. Regressors 13-15 were boxcar regressors that modelled instant signal distortions due to changes in the magnetic field caused by movement of either the mouth or hands. These regressors were therefore not convolved with the HRF. *rightunconv* and *leftunconv* represented distortion due to right and left-hand responses.

CellPress
OPEN ACCESS

They started at the beginning of the TR when the response was recorded and had a duration of one TR (2.28 s). *mouth* represented distortion due to mouth movements. It started at the beginning of the TR when the juice delivery started and terminated at the end of the TR when the juice delivery ended. To further reduce variance and noise in the BOLD signal, we also added task-unrelated confounds which included 13 parametric PCA components that describe, for each volume, the warping from that volume to the mean reference image when correcting motion artefacts. First level analysis was performed on each scanning session (pooled data from both the 'balanced' and 'modified' deigns; 88 scanning session in total). The contrast of parameter estimates (COPEs) and variance estimates (VARCOPEs) from each scanning session were then combined in a second-level mixed-effects analysis (FLAME 1) treating sessions as random effects. Time series statistical analysis was carried out using FMRIB's improved linear model with local autocorrelation correction.

Current Biology, Volume *32*

Supplemental Information

Complementary roles of serotonergic and cholinergic systems in decisions about when to act

Nima Khalighinejad, Sanjay Manohar, Masud Husain, and Matthew F.S. Rushworth

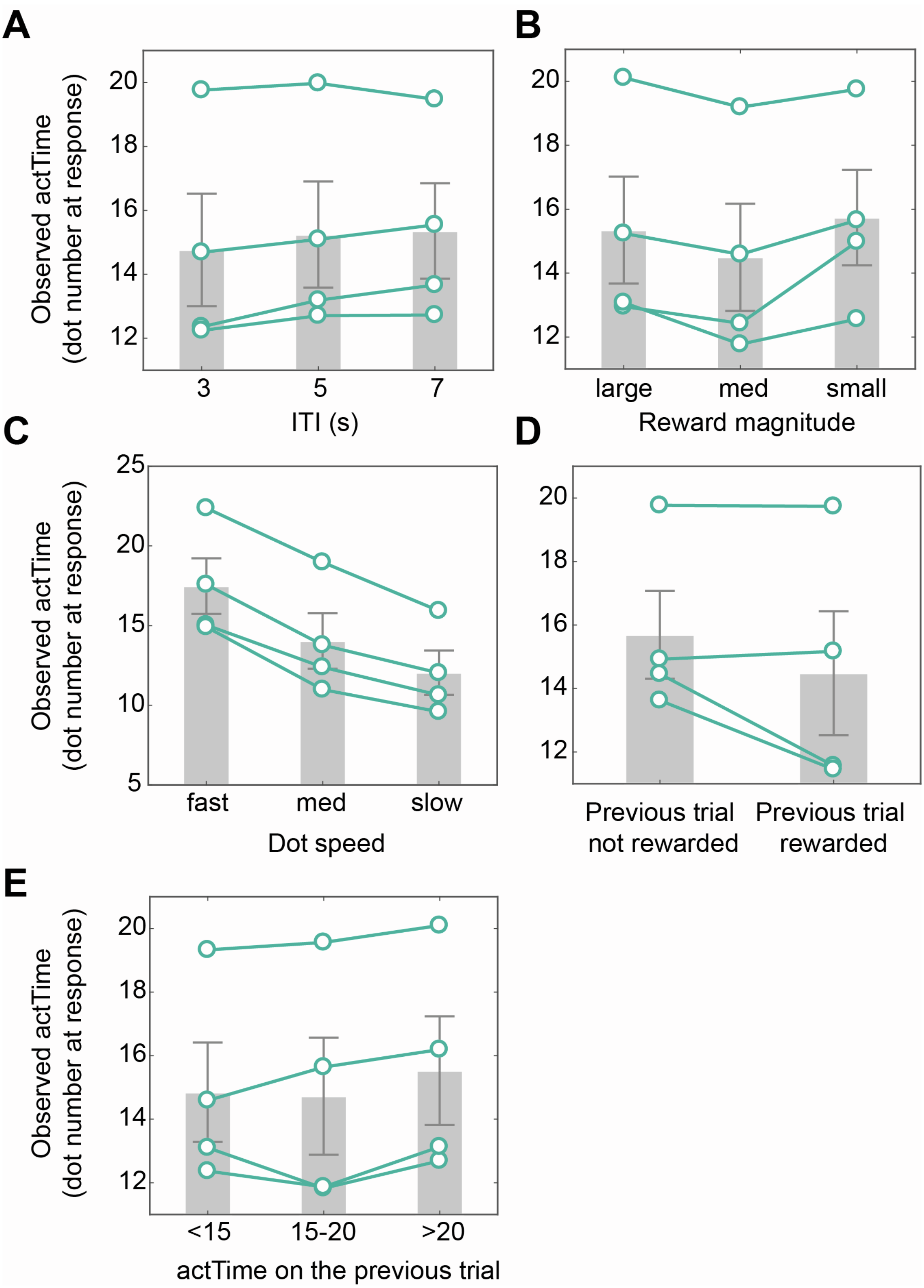
A
Observed actTime
(dot number at response)
ITI (s)
3
5
7
B
Reward magnitude
large
med
small
C
Observed actTime
(dot number at response)
Dot speed
fast
med
slow
D
Previous trial not rewarded
Previous trial rewarded
E
Observed actTime
(dot number at response)
actTime on the previous trial
<15
15-20
>20

Figure S1. The relationship between observed *actTime* and experimentally manipulated variation in the present and recent past contextual factors. Related to Figure 1. This relationship was assessed using the same method as in a previous study[S1]. A multilevel ANOVA (STAR Methods; Khalighinejad et al., 2020) showed that all aspects of present context including ITI (A) ($X^2(2)=17$, $P<0.001$); reward magnitude (B) ($X^2(2)=62$, $P<0.001$); dot speed (C) ($X^2(2)=939$, $P<0.001$); past context including reward outcome on past trial (D) ($X^2(3)=231$, $P<0.001$); and *actTime* on past trial (E) ($X^2(1)=27$, $P<0.001$) influenced animals' *actTime* on the current trial. Post-hoc Tukey's HSD tests showed that animals waited longer before making a response during long compared to short ITI blocks ($\beta=0.14\pm0.03$, $Z=4.14$), when offered a small compared to medium reward ($\beta=0.30\pm0.04$, $Z=8.20$), in fast compared to slow dot speed condition ($\beta=1.24\pm0.03$, $Z=42$), when they had received no reward compared to a large reward on the past trial ($\beta=0.48\pm0.03$, $Z=15$), and when they had already delayed *actTime* on the past trial ($\beta=0.07\pm0.01$). The grey columns are the mean across animals, error bars are the standard error of the mean across animals, and each line is data from individual animals. For illustrative purposes, *actTime* in the immediate past trial is binned into three groups.

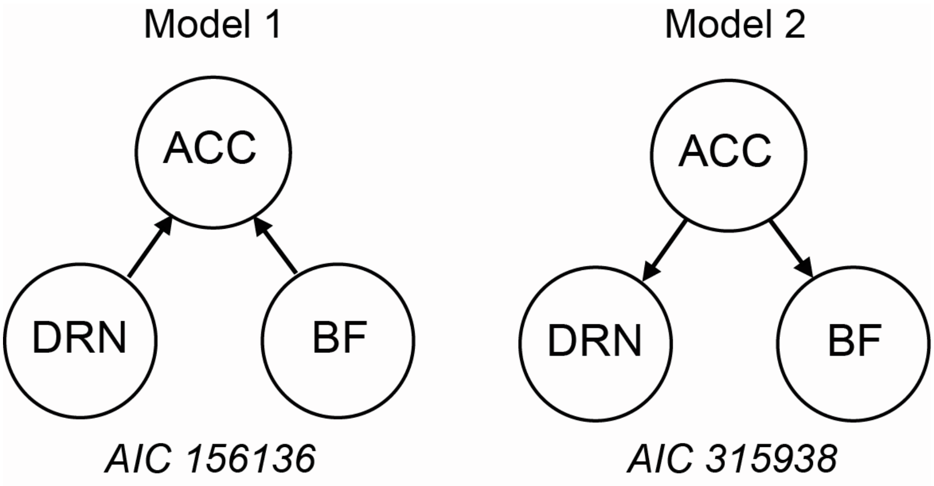

Figure S2. Effective connectivity between ACC, DRN and BF. Related to Figure 3G. Even though PPI analyses demonstrate functional coupling between ROIs as a function of specific psychological measures it does not show directionality. We therefore performed structural equation modelling (SEM) to investigate the direction of the effects. We compared two plausible models: Model 1 has directional connection from DRN and BF to ACC. We did not have any prediction about the directionality of the effect between BF and DRN and therefore did not assume any directionality. Model 2 was similar to Model 1, but all the directions were reversed. SEM showed that a model in which activity in BF and DRN influence ACC (Model1 AIC: 156136) is a better fit to the data than the alternative model (Model2 AIC: 315938).

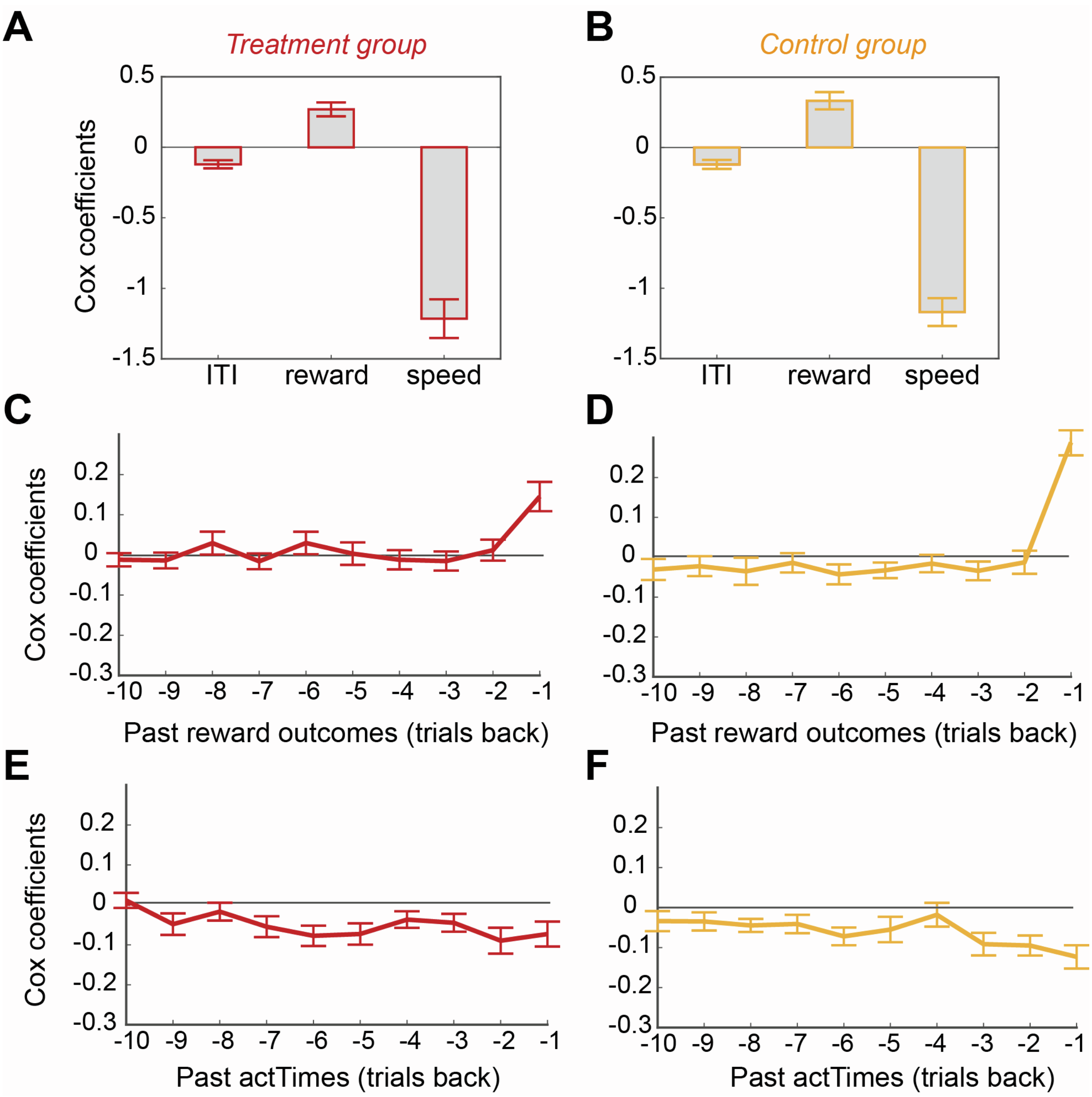
A
Treatment group
Cox coefficients
0.5
0
-0.5
-1
-1.5
ITI
reward
speed
B
Control group
0.5
0
-0.5
-1
-1.5
ITI
reward
speed
C
Cox coefficients
0.2
0.1
0
-0.1
-0.2
-0.3
-10 -9 -8 -7 -6 -5 -4 -3 -2 -1
Past reward outcomes (trials back)
D
0.2
0.1
0
-0.1
-0.2
-0.3
-10 -9 -8 -7 -6 -5 -4 -3 -2 -1
Past reward outcomes (trials back)
E
Cox coefficients
0.2
0.1
0
-0.1
-0.2
-0.3
-10 -9 -8 -7 -6 -5 -4 -3 -2 -1
Past actTimes (trials back)
F
0.2
0.1
0
-0.1
-0.2
-0.3
-10 -9 -8 -7 -6 -5 -4 -3 -2 -1
Past actTimes (trials back)

Figure S3. Coefficients for the covariates used in the Cox regression model. Related to Figure 4. To estimate the fraction of the observed *actTime* that could be predicted from immediate contextual factors we used a Cox proportional hazard model (see STAR Methods). The model predicted time-to-event (*actTime*) on the current trial as a function of the immediate recent past and present context. Specifically, the predictors included reward magnitude, dot speed, and ITI of the current trial (A,B), and the actual reward (C,D) and *actTime* (E,F) on the past 10 trials. The coefficients were estimated separately for each testing session and were used to measure the trial-by-trial variation in *actTime* (i.e., *deterministic actTime*). Serotonergic manipulation had no significant effect on *deterministic actTime* as predicted from the immediate context (Figure 4B). Interestingly, ITI – as compared to other more explicit, stimulus-based features of the environment such as the reward magnitude and dot speed – had the smallest coefficient and therefore a negligible effect on trial-by-trial variation in *deterministic actTime*. This could be due to the fact that ITI – unlike other features of the immediate, present context that varied from trial-to-trial – changed in blocks of 30 trials. This could also explain why ITI's influence on *actTime* and DRN BOLD (see Figures S1 & 5) was comparable to that of the environment's average value (see Figures 1H & 3B). Similarly, the effects of the past reward outcomes and the past *actTimes* were stronger in the immediately recent past trials compared to distant past trials. This further supports our assumption that trial-by trial variation in *actTime* is mostly driven by stimulus-based, immediate, present and recent past context, while ITI and distant past have a more tonic, slow-changing effect on *actTime*, similar to that of the broader, general environment. Error bars are the standard error of the mean across testing sessions.

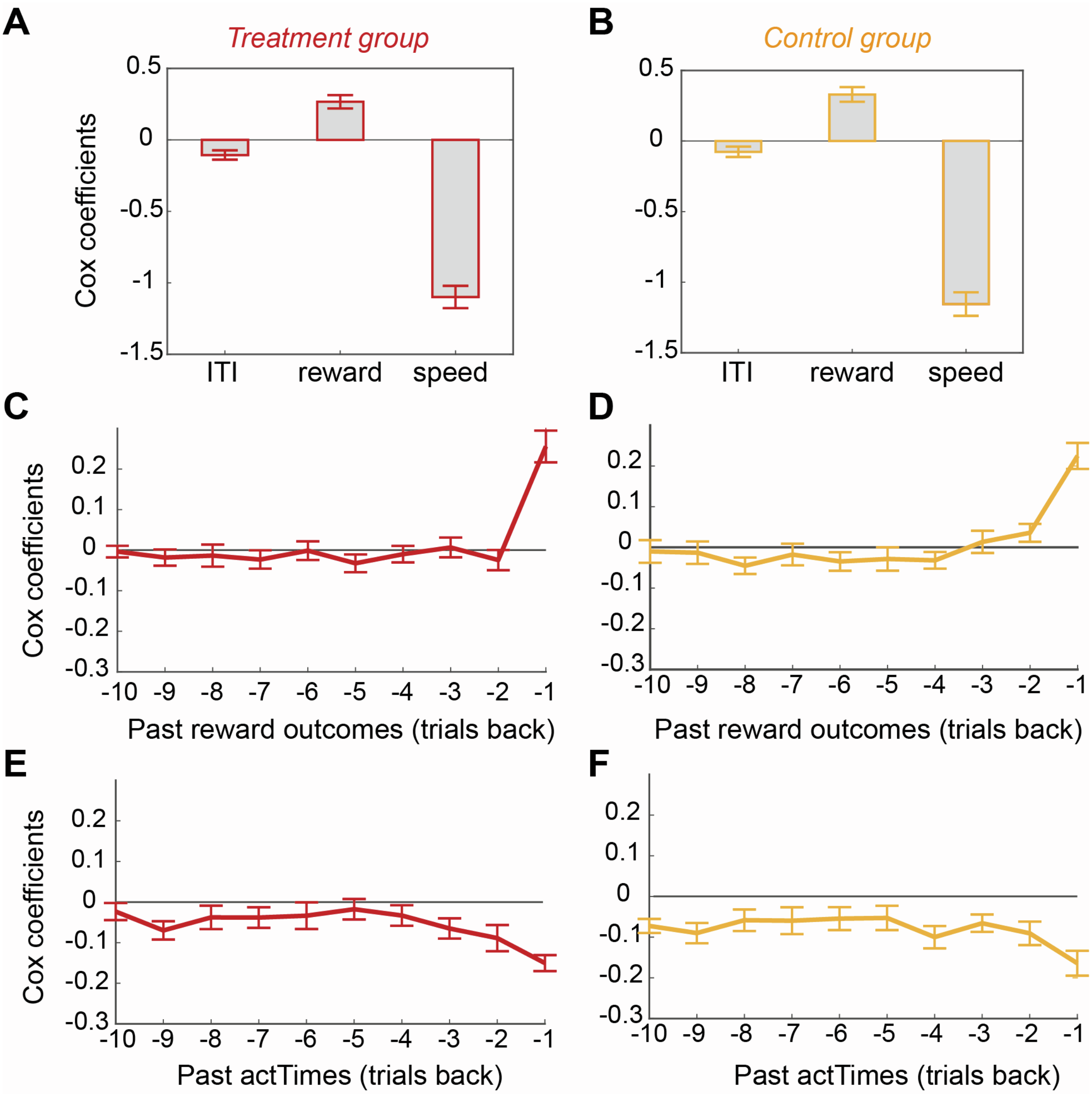

A
Treatment group
B
Control group
Cox coefficients
0.5
0
-0.5
-1
-1.5
ITI
reward
speed
C
D
0.2
0.1
0
-0.1
-0.2
-0.3
-10 -9 -8 -7 -6 -5 -4 -3 -2 -1
Past reward outcomes (trials back)
E
F
Past actTimes (trials back)

Figure S4. Coefficients for the covariates used in the Cox regression model. Related to Figure 6. Similar to Figure S3 but related to data from Exp.3 (cholinergic manipulation). The coefficients were estimated separately for each testing session and were used to measure the trial-by-trial variation in *actTime* (i.e., *deterministic actTime*). Cholinergic manipulation significantly reduced the length of time each animal was expected to wait on each trial before making a response, as predicted from the combined effect of the immediate recent past and present context (i.e., *deterministic actTime;* Figure 6B). However, comparing the coefficients between the Treatment and the Control groups showed that while the combined effect of the contextual factors reduced *deterministic actTime* in the Treatment group, this effect was not mediated by any one particular factor. This is consistent with the interaction effects in GLM3.1. Additionally, looking at Figure 6 it seems that in one particular animal (Monkey C) the effect of cholinergic manipulation has opposite effects on observed and deterministic *actTime*. We think this difference is due to two reasons: First, it stems from the relatively high number of un-responded trials in Monkey C (Figure 6C; %30). This is problematic for the observed *actTime* analysis because it leads to a reduction in data. However, it is less problematic for the deterministic *actTime* analysis because these missed trials are modelled as censored data in the Cox regression model (i.e., the event time exceeds the censoring time). Second, the deterministic *actTime* in Monkey C has an ex-Gaussian distribution with a relatively long tail, similar to distribution of the observed and deterministic *actTime* in other three monkeys, but different from the large-width Gaussian distribution of observed *actTime* in the same monkey. The effect of cholinergic manipulation on *actTime* could therefore be more easily identified as a change in the mean of the Gaussian component of the ex-Gaussian distribution in this monkey. It thus became clear, when using this approach, that a similar change had occurred in all four animals under the cholinergic manipulation. Error bars are the standard error of the mean across testing sessions.

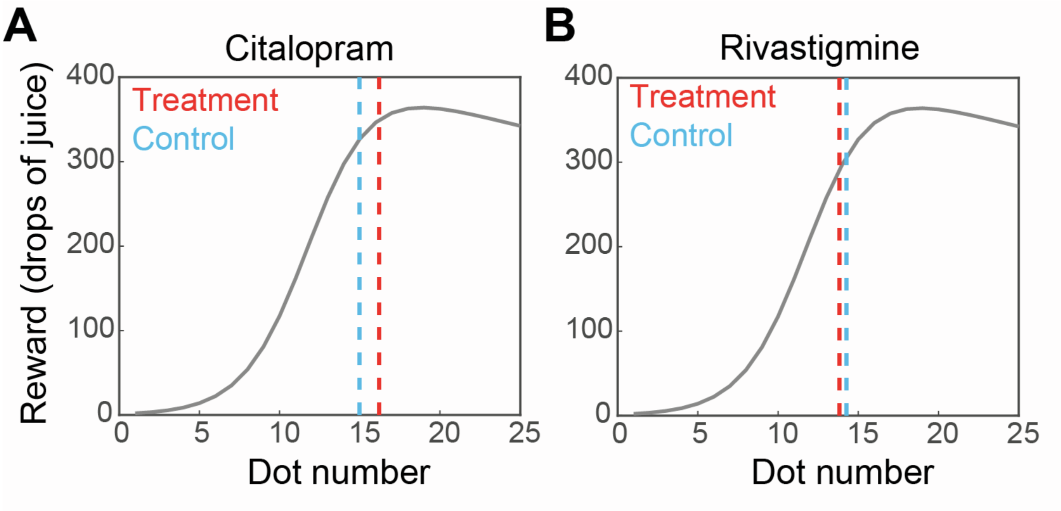

Figure S5. The effect of citalopram and rivastigmine on accumulated reward. Related to Figures 4&6. While our approach does not provide a normative account of animals' decisions it is possible to estimate the best speed on which to respond given the various task features such as the sigmoid reward function (Figure 1C) and the total length of each session (40 minutes). This shows that monkeys would collect the highest amount of reward (drops of juice) if they waited for 19 dots before responding. In the Citalopram study (A) monkeys on average waited for 16.2 dots before responding in the Treatment condition (red dashed line) and 15.2 dots in the Control condition (blue dashed line). This resulted in monkeys collecting significantly more reward in the Treatment compared to the Control group ($t(19)=2.36$, $P=0.029$). When we make an analogous comparison in the Rivastigmine study (B), however, we found no significant difference between the reward rates in the Treatment and the Control conditions ($P=0.51$). This suggests that in healthy animals increasing levels of ACh does not make the behaviour more optimal. However, in pathologies such as Parkinson's disease, which is often associated with apathy, rivastigmine might help to alleviate symptoms by invigorating volitional movements in response to environmental stimuli.

The slowing of *actTime* induced by SSRI administration led the animals to respond more closely to the optimum time and so they obtained more rewards in total. Some of the changes in *actTime* that occurred when the task environment was changed in the absence of any pharmacological treatment may also have been adaptive. For example, macaques responded more deliberately and slowly during long compared to short ITI blocks when the rate of reward was lower than the average reward rate elsewhere in the same day's testing session; careful, long *actTimes* ensure opportunities to obtain reward are not wasted before a long transition to the next trial. However, the finding that animals responded more slowly on medium value trials that occurred in the context of high average value, is not clearly

adaptive in the same way. Slow responses on such trials entail an opportunity cost; the opportunity costs of acting slowly are higher when the offer is worth less than the average value of the environment. A full account of how and why changes in reward distributions lead to changes in vigour and speed of responding remains elusive.

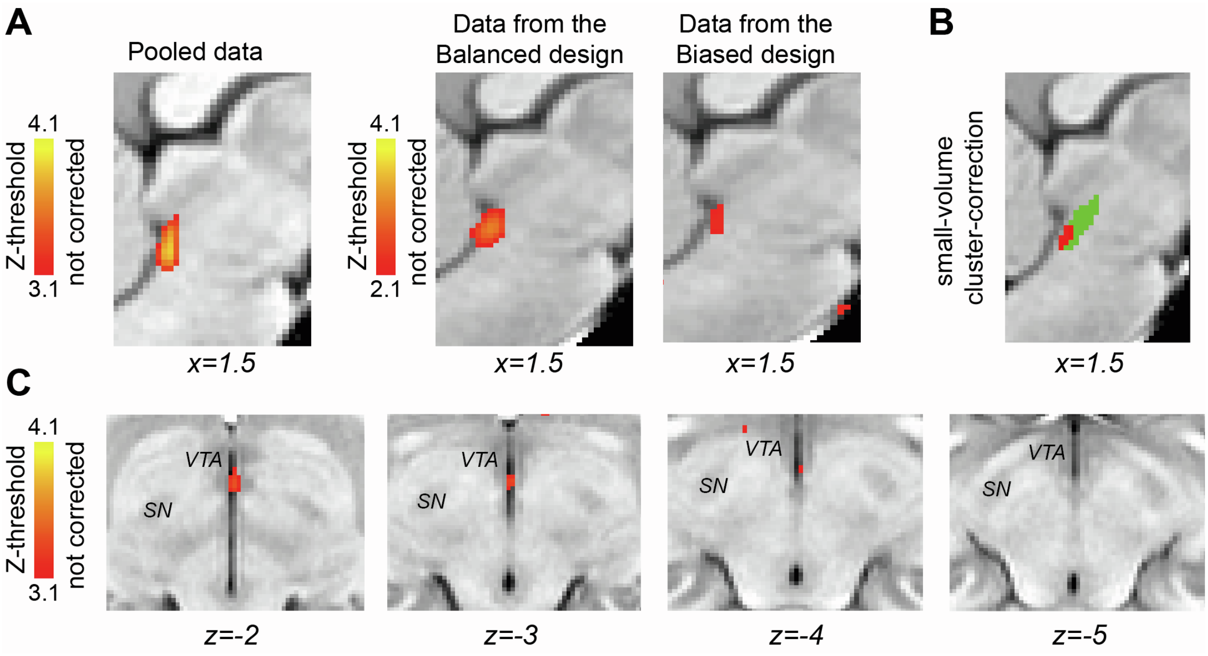

Figure S6. The relationship between BOLD and ITI. Related to Figure 5. (A) Correlation between DRN BOLD and ITI shown separately for the pooled data, data from the balanced design, and data from the biased design. the ITI effect at DRN was weaker when looking at the datasets separately (because each analysis employs approximately half as much data as is the case when the data sets are combined) and could only be detected at lower Z-thresholds (Z>2.1). Nevertheless, in both datasets we found a positive correlation between DRN voxels and ITI. (B) The effect of ITI from the DRN region that was used in Exp.1 for the analysis of the average value. The green mask shows the DRN region that was used in Exp.1. The overlapping red mask shows the cluster with a significant ITI effect (Z-threshold=3.1; peak Z=3.82, Caret-F99 Atlas (F99): x=1.0, y=-21, z=-8.5; small-volume correction; number of voxels=31, P=0.003). (C) The volume of interest that we used in Figure 5 already covered the midbrain including the dopaminergic structures that are often associated with encoding of the reward rate including the ventral tegmental area (VTA) and the substantia nigra (SN). Having found no significant cluster other than DRN thus suggests that dopaminergic midbrain activity is not significantly correlated with ITI. However, it is still possible that without cluster correction we might detect an ITI effect in dopaminergic midbrain. Therefore, without performing cluster correction, we searched for voxels within VTA/SN but could not find any with significant positive correlation with ITI (Z>3.1).

	Monkey 1	Monkey 2	Monkey 3	Monkey 4
Phase I				
**Day 1-7 (10mg)**	*Placebo*	*Placebo*	*Citalopram*	*Citalopram*
**Day 8-14 (20mg)**	*Placebo*	*Placebo*	*Citalopram*	*Citalopram*
**Day 15-24 (20mg)**	*Placebo*	*Placebo*	*Citalopram*	*Citalopram*
**5-HT measurement (nmoles/L)**	1519	1780	70	103
**Day 25-38 (Wash-out)**				
Phase II				
**Day 39-45 (10mg)**	*Citalopram*	*Citalopram*	*Placebo*	*Placebo*
**Day 46-52 (20mg)**	*Citalopram*	*Citalopram*	*Placebo*	*Placebo*
**Day 53-62 (20mg)**	*Citalopram*	*Citalopram*	*Placebo*	*Placebo*
**5-HT measurement (nmoles/L)**	160	280	1863	1137

Table S1. Citalopram dosing schedule. Related to Figure 4. Behavioural data collection for the main analyses was conducted on alternate days during the last 10 days (green cells). Groups were switched after a 2-week wash-out period. 5-HT levels were measured in the platelet rich plasma (PRP) after each treatment phase (within-subject comparison). Note that selective serotonin reuptake inhibitors block serotonin receptors (SERT) on platelets, thereby preventing reuptake of 5-HT into platelets. The results clearly show that concentration of 5-HT within platelets decreased when monkeys were in the treatment compared to the control group. We also collected blood samples after ACh treatment to check that the manipulations were working. However, measurement of ACh levels for all individuals was not possible because of the impact of COVID-19-related lockdown in the UK.

	Monkey 1	Monkey 2	Monkey 3	Monkey 4
Phase I				
**Day 1-7 (0.37mg)**	*Rivastigmine*	*Rivastigmine*	*Placebo*	*Placebo*
**Day 8-14 (0.75mg)**	*Rivastigmine*	*Rivastigmine*	*Placebo*	*Placebo*
**Day 15-24 (1.5mg)**	*Rivastigmine*	*Rivastigmine*	*Placebo*	*Placebo*
**Day 25-38 (Wash-out)**				
Phase II				
**Day 39-45 (0.37mg)**	*Placebo*	*Placebo*	*Rivastigmine*	*Rivastigmine*
**Day 46-52 (0.75mg)**	*Placebo*	*Placebo*	*Rivastigmine*	*Rivastigmine*
**Day 53-62 (1.5mg)**	*Placebo*	*Placebo*	*Rivastigmine*	*Rivastigmine*

Table S2. Rivastigmine dosing schedule. Related to Figure 6. Behavioural data collection for the main analyses was conducted on alternate days during the last 10 days (green cells). Groups were switched after a 2-week wash-out period.

Supplemental References

S1. Khalighinejad, N., Bongioanni, A., Verhagen, L., Folloni, D., Attali, D., Aubry, J.-F., Sallet, J., and Rushworth, M.F.S. (2020). A Basal Forebrain-Cingulate Circuit in Macaques Decides It Is Time to Act. Neuron *105*, 370-384.e8.
